# Pan‐European phylogeography of the European roe deer (*Capreolus capreolus*)

**DOI:** 10.1002/ece3.8931

**Published:** 2022-05-19

**Authors:** Kamila Plis, Magdalena Niedziałkowska, Tomasz Borowik, Johannes Lang, Mike Heddergott, Juha Tiainen, Aleksey Bunevich, Nikica Šprem, Ladislav Paule, Aleksey Danilkin, Marina Kholodova, Elena Zvychaynaya, Nadezhda Kashinina, Boštjan Pokorny, Katarina Flajšman, Algimantas Paulauskas, Mihajla Djan, Zoran Ristić, Luboš Novák, Szilvia Kusza, Christine Miller, Dimitris Tsaparis, Stoyan Stoyanov, Maryna Shkvyria, Franz Suchentrunk, Miroslav Kutal, Vukan Lavadinović, Dragana Šnjegota, Ana‐Maria Krapal, Gabriel Dănilă, Rauno Veeroja, Elżbieta Dulko, Bogumiła Jędrzejewska

**Affiliations:** ^1^ Mammal Research Institute Polish Academy of Sciences Białowieża Poland; ^2^ Working Group for Wildlife Research at Clinic for Birds, Reptiles, Amphibians and Fish Justus‐Liebig‐University Giessen Gießen Germany; ^3^ Department of Zoology Musée National d’Histoire Naturelle Luxembourg City Luxembourg; ^4^ 419837 Lammi Biological Station University of Helsinki Lammi Finland; ^5^ Natural Resources Institute Finland (Luke) Helsinki Finland; ^6^ State National Park Belovezhskaya Pushcha Kamenyuki Republic of Belarus; ^7^ Department of Fisheries, Apiculture, Wildlife Management and Special Zoology Faculty of Agriculture University of Zagreb Zagreb Croatia; ^8^ Department of Phytology Technical University in Zvolen Zvolen Slovak Republic; ^9^ 54744 A.N. Severtsov Institute of Ecology and Evolution Russian Academy of Sciences Moscow Russia; ^10^ 68910 Faculty of Environmental Protection Velenje Slovenia; ^11^ 68910 Department of Forest Ecology Slovenian Forestry Institute Ljubljana Slovenia; ^12^ 105468 Department of Biology Vytautas Magnus University Kaunas Lithuania; ^13^ Department of Biology and Ecology Faculty of Sciences University of Novi Sad Novi Sad Republic of Serbia; ^14^ Department of Geography, Tourism and Hotel Management Faculty of Sciences University of Novi Sad Novi Sad Serbia; ^15^ 48269 Department of Forest Protection and Wildlife Management Mendel University in Brno Brno Czech Republic; ^16^ 37599 Centre for Agricultural Genomics and Biotechnology Faculty of Agricultural and Food Sciences and Environmental Management University of Debrecen Debrecen Hungary; ^17^ Bureau of Wildlife Biology Bavaria Rottach‐Egern Germany; ^18^ Institute of Marine Biology, Biotechnology and Aquaculture (IMBBC) Hellenic Centre for Marine Research Heraklion, Crete Greece; ^19^ 185985 Wildlife Management Department University of Forestry Sofia Bulgaria; ^20^ Kyiv Zoological Park of National Importance Kyiv Ukraine; ^21^ Research Institute of Wildlife Ecology University of Veterinary Medicine Vienna Vienna Austria; ^22^ 48269 Department of Forest Ecology Faculty of Forestry and Wood Technology Mendel University in Brno Brno Czech Republic; ^23^ Faculty of Forestry University of Belgrade Belgrade Serbia; ^24^ 186630 Faculty of Natural Sciences and Mathematics University of Banja Luka Banja Luka Bosnia and Herzegovina; ^25^ "Grigore Antipa" National Museum of Natural History Bucharest Romania; ^26^ Faculty of Forestry Stefan cel Mare University of Suceava Suceava Romania; ^27^ Department of Wildlife Monitoring Estonian Environment Agency Tallin Estonia; ^28^ 49605 Department of Anesthesiology University of Virginia Health System Charlottesville Virginia USA; ^29^ 49605 Faculty of Biology University of Warsaw Warszawa Poland

**Keywords:** *Capreolus capreolus*, expansion, mitochondrial DNA, the Last Glacial Maximum refugia, the Quaternary history

## Abstract

To provide the most comprehensive picture of species phylogeny and phylogeography of European roe deer (*Capreolus capreolus*), we analyzed mtDNA control region (610 bp) of 1469 samples of roe deer from Central and Eastern Europe and included into the analyses additional 1541 mtDNA sequences from GenBank from other regions of the continent. We detected two mtDNA lineages of the species: European and Siberian (an introgression of *C*. *pygargus* mtDNA into *C*.* capreolus*). The Siberian lineage was most frequent in the eastern part of the continent and declined toward Central Europe. The European lineage contained three clades (Central, Eastern, and Western) composed of several haplogroups, many of which were separated in space. The Western clade appeared to have a discontinuous range from Portugal to Russia. Most of the haplogroups in the Central and the Eastern clades were under expansion during the Weichselian glacial period before the Last Glacial Maximum (LGM), while the expansion time of the Western clade overlapped with the Eemian interglacial. The high genetic diversity of extant roe deer is the result of their survival during the LGM probably in a large, contiguous range spanning from the Iberian Peninsula to the Caucasus Mts and in two northern refugia.

## Graphical abstract

Among European ungulates, the roe deer (*Capreolus capreolus*) is the most widely distributed species and we decided to investigate how the past features impacted the current genetic patterns of this species. With the approach of the large‐scale sampling, we decided to fill gaps in knowledge and prepare the most comprehensive study about roe deer genetic in Europe. We also took an attempt to define possible areas, important for the species survival during Last Glacial Maximum.

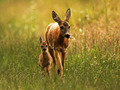

## 1 INTRODUCTION

Climate fluctuations during the Last Glacial Period between 115,000 and 11,500 years ago (Lokrantz & Sohlenius, 2006) played an important role in shaping the current species composition, distribution, and genetic diversity of mammals in Europe. During glaciations, the ranges of cold sensitive species were limited to refugial areas located in the Balkan, the Iberian and the Apennine Peninsulas (Hewitt, 2004; Taberlet et al., 1998) and south‐eastern part of the continent (the Black Sea region and the Caucasus Mts.; Markova & Puzachenko, 2019). Contribution of a given refugium into postglacial recolonization processes varied a lot among species such as, for example, red deer *Cervus elaphus* (Niedziałkowska, Doan, et al., 2021), wild boar *Sus scrofa* (Niedziałkowska, Tarnowska, et al., 2021), or common vole *Microtus arvalis* (Stojak et al., 2015). Cold‐adapted species such as, for example, reindeer *Rangifer tarandus* (Sommer et al., 2014), saiga antelope *Saiga tatarica* (Nadachowski et al., 2016), or arctic fox *Alopex lagopus* (Dalén et al., 2007), thrived in the glacial stages, expanded their ranges southward, and during the onset of interglacial they underwent massive, large‐scale extinctions. Species of mammals that have very broad biogeographic niche (from the Mediterranean to the boreal zone) had more diverse response to geological time‐scale pulsation of climate, most likely with several glacial refugia located at both lower and higher latitudes, where subpopulations diverged into different lineages and could have developed adaptations to different climate, habitat, and food‐related conditions. Examples of such species include the bank vole *Clethrionomys glareolus* (Tarnowska et al., 2019), weasel *Mustela nivalis* (McDevitt et al., 2012), common vole *M. arvalis*, and field vole *Microtus agrestis* (Baca et al., 2020; Stojak et al., 2019).

**FIGURE 1:**
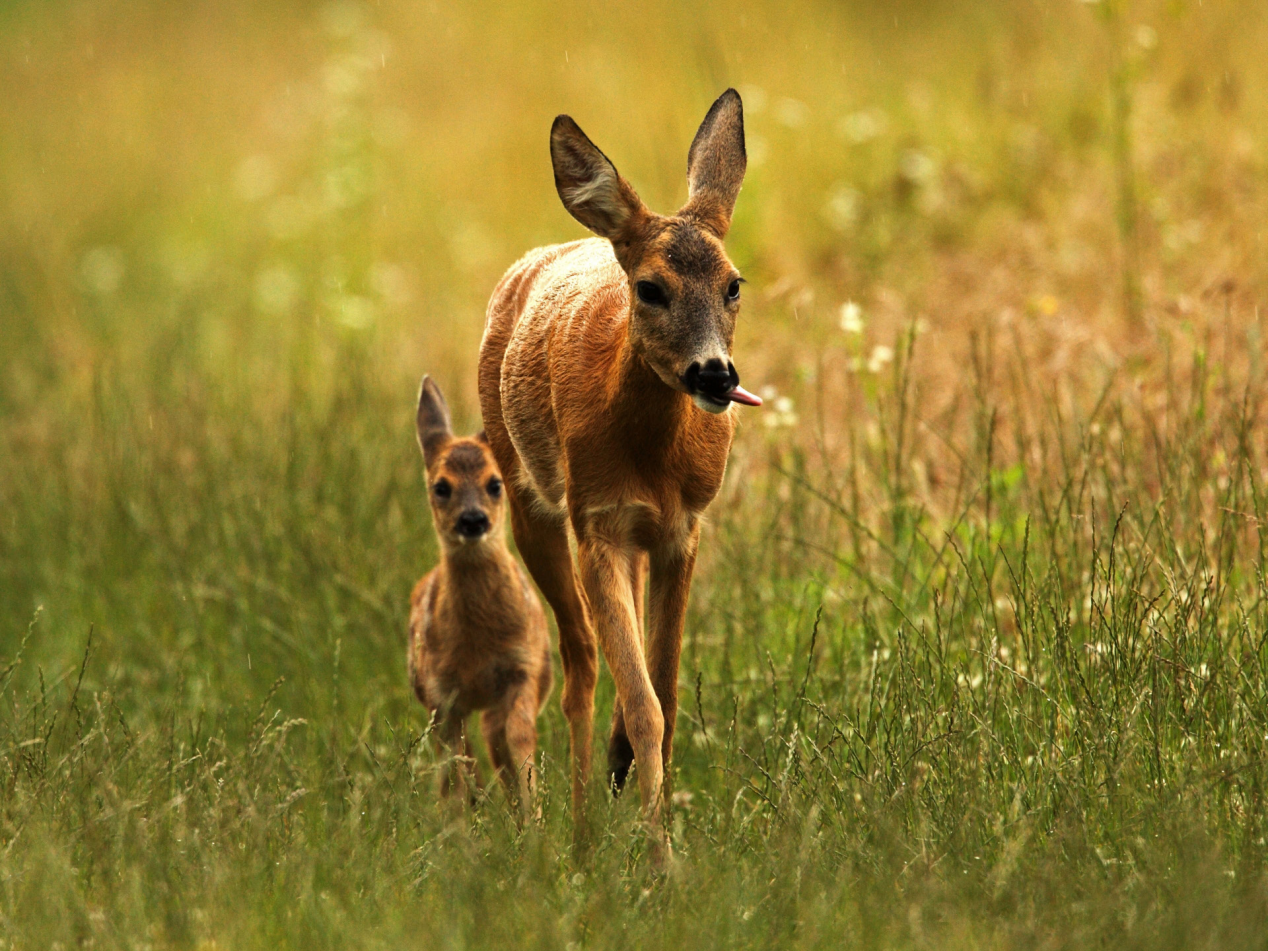
Roe deer female with fawn. Photograph by Tibor Pataky

**FIGURE 2:**
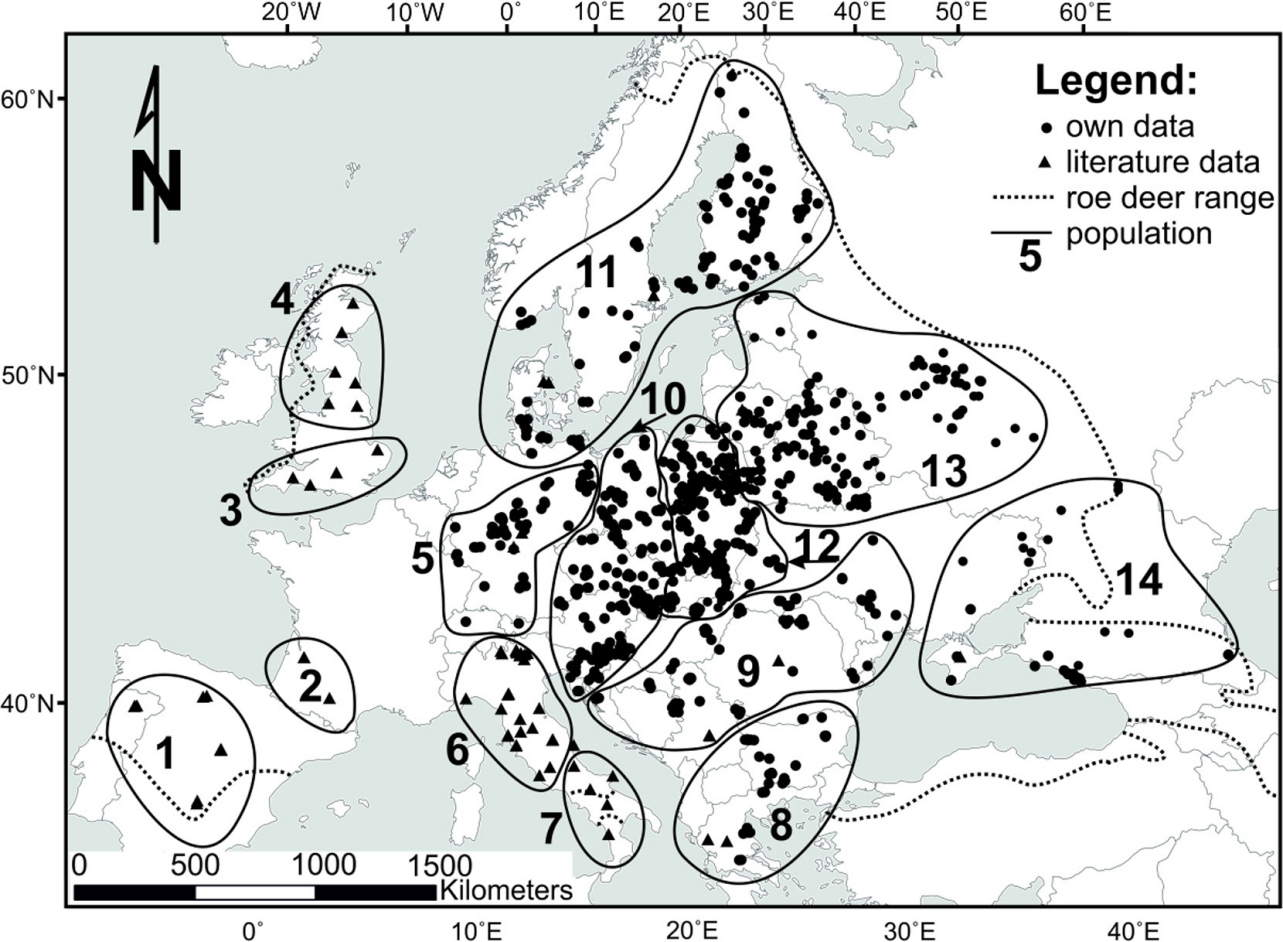
Roe deer (*Capreolus capreolus*) sampling locations in Europe: distribution of own and literature data in the species range

Among European ungulates, the roe deer (*Capreolus capreolus* Linnaeus, 1758) (Figure 1) is the most widely distributed species (Lovari et al., 2016). It occurs throughout the continent (Figure 2), except for the northernmost Scandinavia and some islands. In southern Europe, roe deer lives in the Mediterranean zone, and the eastern range of the species reaches the Caucasus Mountains, northern Iran, and Iraq (Danilkin, 1995). The eastern border of its range runs through western Russia. Roe deer is a habitat opportunist occupying both forests and open habitats (Apollonio et al., 2010; Geist, 1998). European roe deer is distinctly smaller that the Siberian roe deer (*Capreolus pygargus* (Pallas, 1771) occurring in Asia and in the south of the European part of Russia. The present ranges of the two species overlap in the Volga‐Don region (Kashinina et al., 2018).

In some areas, European roe deer is an important game species. In historical times, when the populations were locally exterminated or their numbers were low, there were attempts to strengthen them with introduced animals (Apollonio et al., 2014; Baker & Hoelzel, 2013).

So far, phylogenetic studies on European roe deer covered a large part of their contemporary range and indicated three main mitochondrial DNA (mtDNA) clades—Central, Eastern, and Western (Baker & Hoelzel, 2014; Lorenzini et al., 2014; Randi et al., 2004; Tsaparis et al., 2019). The Central clade is the most common throughout Europe, the Eastern one is restricted mainly to the Balkans, and the Western clade occurs only in the Iberian Peninsula (Gentile et al., 2009; Lorenzini et al., 2014; Mucci et al., 2012; Randi et al., 2004). In addition, on the basis of internal structuring within clades, some researchers proposed the separation of the roe deer occurring in Italy as a distinct subspecies *C*. *c*. *italicus* (Gentile et al., 2009; Mucci et al., 2012). In Spain, there were also attempts to identify the Celtic‐Iberian mtDNA clade (Baker & Hoelzel, 2014; Royo et al., 2007) and a subspecies *C*. *c*. *garganta* (Meunier, 1983).

Recent studies showed the introgression of the Siberian roe deer mtDNA genes into the eastern populations of the European roe deer (Kashinina et al., 2018; Lorenzini et al., 2014; Markov et al., 2016; Matosiuk et al., 2014). This showed a new level of the genetic complexity in the species and opened a debate on the sources of that admixture (Kashinina et al., 2018; Lorenzini et al., 2014; Matosiuk et al., 2014; Świsłocka et al., 2019). However, the limited sampling (Lorenzini et al., 2014) did not allow to define whole spatial range at which the introgression has occurred.

Despite multiple studies on European roe deer phylogeography, there still remain substantial gaps in the sampling coverage in Europe, which make the picture incomplete and thus inconclusive. There were no roe deer studies done in northern (Finland), eastern (Belarus, Estonia), central (Slovakia, Czech Republic), and south‐eastern (Croatia, Slovenia) parts of the continent. The most comprehensive phylogeographical analyses, done by Lorenzini et al. (2014) and Randi et al. (2004) focused mostly on identifying the origin of mtDNA clades and on disentangling the relationships among them. So far, there had been no attempts to determine the location of the contact zones between them. Moreover, the broad spatial spread of the clades suggests that, apart from separation of the *C*. *c*. *italicus* subspecies, there is some internal heterogeneity within the three main clades.

In our study, we aimed at reconstructing the phylogeny of roe deer on the basis of intensive sampling in central and Eastern Europe, including many areas that have not been studied before. We pooled our new data on mtDNA sequences with already available roe deer sequences in order to perform a more detailed and comprehensive analysis of roe deer phylogeography in the whole European range of the species (in 26 countries). The aims of our study, based on the analysis of a 610‐bp fragment of mtDNA control region, were to: (1) describe the genetic diversity of roe deer over the entire species range; (2) identify spatial patterns of the clade distribution; and (3) compare the demographic processes at the level of lineages, clades, and haplogroups. Finally, we discuss the possible LGM refugia and routes of postglacial recolonization of the continent by roe deer.

## 2 MATERIAL AND METHODS

### 2.1 Sample and data collection

During 2008–2017, we collected 1469 roe deer samples from 20 countries in central and Eastern Europe (Table 1, Figure 2). The study area ranged from Germany to Russia (6°35′–43°23′E) and from Finland to Greece (67°42′–38°44′N). Samples, which consisted of fresh fragments of skin or muscle from legally hunted animals, were in majority taken by hunters. Prior to DNA extraction, all samples were stored in 96% ethanol at –20°C. We assigned the geographic coordinates of the samples on the basis of information on location provided by the hunters. Additionally, we included 1541 sequences of roe deer from western and southern Europe (see Table S1), based on data available in GenBank and their frequencies reported in publications (Baker & Hoelzel, 2013; Biosa et al., 2015; Gentile et al., 2009; Lorenzini et al., 2014; Randi et al., 2004; Royo et al., 2007).

**TABLE 1:** The number of roe deer *Capreolus capreolus* samples analyzed from the European countries

Country	Number of samples			
	This study	Literature	Total	
Austria	16	6	22	Lorenzini et al. (2014)
Belarus	135	–	135	–
Bulgaria	42	–	42	–
Croatia	25	–	25	–
Czech Republic	58	–	58	–
Denmark	–	19	19	Lorenzini et al. (2014), Randi et al. (2004)
Estonia	5	–	5	–
Finland	106	–	106	–
France	–	24	24	Lorenzini et al. (2014), Randi et al. (2004)
Germany	159	15	174	Randi et al. (2004)
Greece	52	15	67	Lorenzini et al. (2014), Randi et al. (2004)
Hungary	54	–	54	–
Italy	–	747	747	Biosa et al. (2015), Gentile et al. (2009), Lorenzini et al. (2014), Randi et al. (2004)
Lithuania	16	13	29	Lorenzini et al. (2014)
Norway	7	–	7	–
Poland	382	8	390	
Portugal	–	23	23	Randi et al. (2004)
Romania	15	10	25	Lorenzini et al. (2014)
Russia	97	–	97	–
Serbia	50	178	228	Randi et al. (2004)
Slovakia	99	–	99	–
Slovenia	65	–	65	–
Spain	–	156	156	Lorenzini et al. (2014), Randi et al. (2004)
Sweden	17	11	28	Lorenzini et al. (2014), Randi et al. (2004)
Ukraine	69	3	72	Lorenzini et al. (2014)
United Kingdom	–	313	313	Baker and Hoelzel (2013)
Total	1469	1541	3010	

### 2.2 DNA extraction and sequencing

We extracted total genomic DNA using the Qiagen DNeasy Blood and Tissue Kit following the manufacturer’s guidelines. A fragment of the mtDNA control region was amplified by PCR with the primers L‐Pro and H‐Phe (Randi et al., 1998). Cycling conditions were 95°C for 15 min; 35 cycles of 94°C for 15 s, 56°C for 15 s, and 72°C for 1 min; and a final step of 72°C for 10 min. PCR products were purified using Clean Up (A&A Biotechnology). Sequencing reactions were carried out in a 10 µl volume using the Big Dye sequencing kit v.3.1 (Applied Biosystems) with the forward primer. Products were purified with the Exterminator kit (A&A Biotechnology) and sequenced on an ABI 3130 xl Genetic Analyzer (Applied Biosystems). Sequencing results were analyzed with the ABI DNA Sequencing Analysis software and aligned in BioEdit v.7.0.5.3 (Hall, 1999).

### 2.3 Sequence and data analyses

Sequencing resulted in good‐quality mitochondrial control region fragments of 610 bp for all analyzed samples. Further, these fragments were aligned against the sequence of European roe deer from the north‐eastern Poland (GenBank KM068161.1; Olano‐Marin et al., 2014) and manually edited in BioEdit v.7.0.5.3 Hall (1999). Additional roe deer mtDNA data from GenBank were pooled with the obtained own fragments and shortened to keep the same length for all sequences.

We assigned the obtained sequences to haplotypes using Arlequin 3.5.1.3 software (Excoffier & Lischer, 2010). Indels were considered as differences in haplotype definition. Each haplotype was assigned a code reflecting one of the two species: Cc—for the European roe deer (*C*. *capreolus*) or Cp—for the Siberian roe deer (*C*. *pygargus*). Internal structure of haplogroups was based on haplotype genealogy constructed in HapView (Salzburger et al., 2011). Italian haplogroup *C*. *c*. *italicus* (C7 in this study) has already been defined as such in the literature (Gentile et al., 2009; Mucci et al., 2012).

To determine sequence evolution model, we used jModelTest2 (Darriba et al., 2012), using resources available on CIPRES Science Gateway (Miller et al., 2010). The best model chosen by jModelTest2 was the Hasegawa, Kishino, and Yano model (HKY; Hasegawa et al., 1985) with additional variation rate among sites and a proportion of invariable sites (+G, +I). Phylogenetic trees were constructed in Mega 7.0.14 (Kumar et al., 2016) and MrBayes 3 (Ronquist & Huelsenbeck, 2003) with the maximum likelihood method and 10,000 bootstrap replications, using the chosen model. Summary statistics were calculated in DnaSP 5.10.01 (Librado & Rozas, 2009) according to three classification levels: lineage, clade, and grouping based on HapView. The following statistics were computed: number of unique haplotypes (*h*), number of segregating (polymorphic) sites (*S*), haplotype diversity (*H*_d_), nucleotide diversity (*π*), and average number of pairwise nucleotide differences (*k*). Additionally we included *B* index (Levins, 2020) to express the diversity of haplotypes, using the formula:

$$B=\;\frac{1}{\sum p_{i}^{2}}$$

where *p_i_* is the proportion of samples with haplotype *i* in a deme. The *B* index minimum value is 1 and its upper bound is equal to the number of haplotypes in the sample.

To evaluate possible models of expansion, we performed two neutrality tests in DnaSP: Tajima's *D* (Tajima, 1989) and Fu's Fs (Fu, 1997). We also used mismatch distribution with the sudden expansion model and goodness‐of‐fit tests (sum of squared deviation; Harpending's raggedness index (*R*). Expansion time (*T*) was estimated based on equation: *T* = *τ* /2*µ*, where *τ* is Tau estimated in Arlequin 3.5.1.3 and *µ* is the mutation rate described as units of substitutions per locus per generation (Rogers & Harpending, 1992). We applied a mutation rate of 0.04–0.08 substitutions per site per Myr (Lorenzini et al., 2014; Randi et al., 1998, 2004; Royo et al., 2007; Vernesi et al., 2002) and used 3 years as a generation time (Randi et al., 1998, 2004). Time since expansion was calculated using the Excel Spreadsheet provided by Schenekar and Weiss (2011).

The Last Glacial Period had impact on large areas of the northern hemisphere and its names differ regionally. Throughout this manuscript we used the name “Weichselian” as reference, which is equivalent to the other terms such as “Vistulian” (van Loon et al., 2012), “Wurm” or “Devensian” (Kolfschoten, 2000). The reconstruction of the potential LGM refugial range of European roe deer during that period was based on fossil and subfossil records of the species provided by Sommer et al. (2009) and Markova and Puzachenko (2019), supplemented with habitat availability data (Markova & Puzachenko, 2019).

## 3 RESULTS

### 3.1 Phylogenetics of roe deer

**FIGURE 3:**
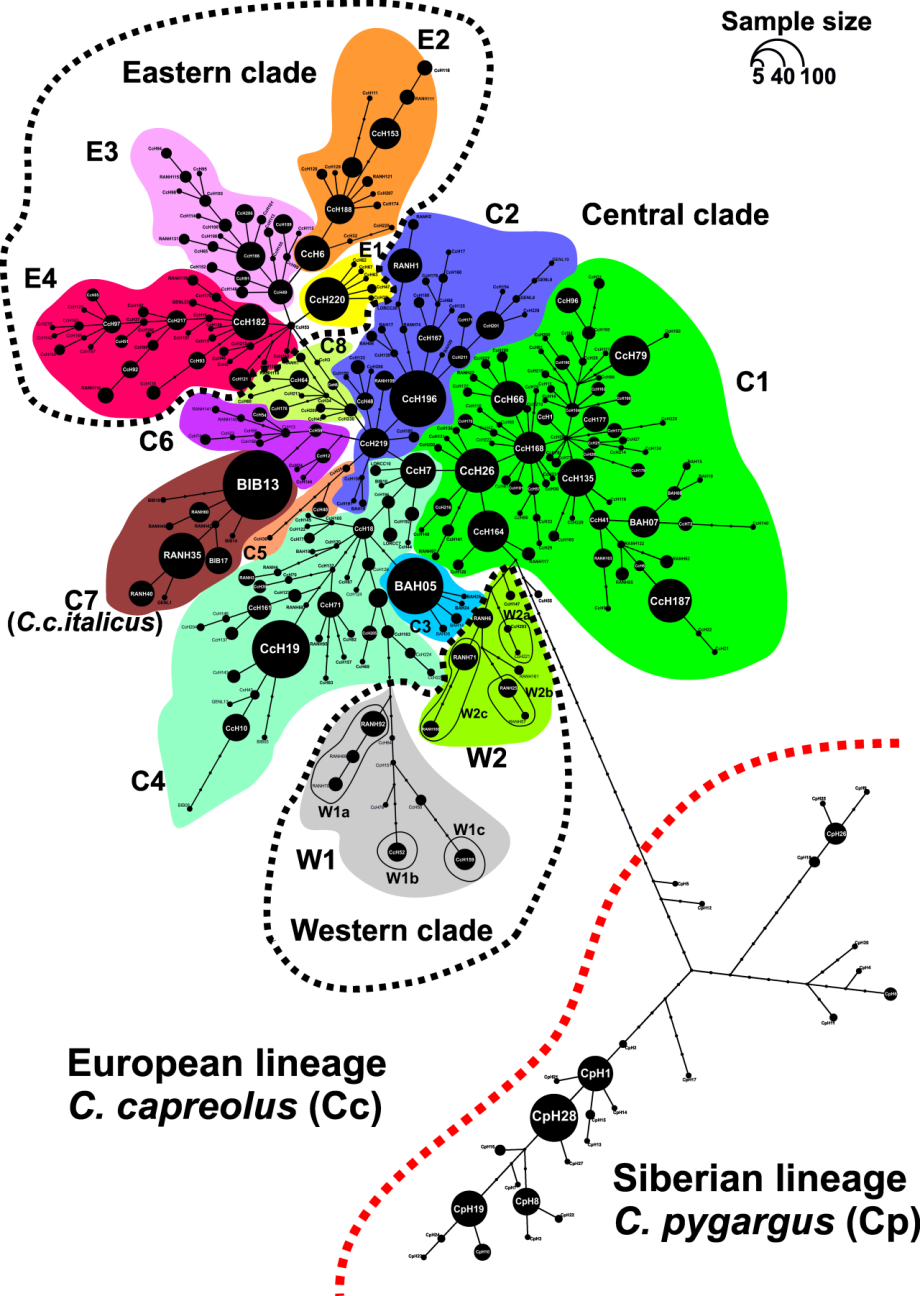
Internal structure of European roe deer (*C*. *capreolus*) clades defined based on mtDNA haplotype genealogy constructed in HapView program. Circles represent unique haplotypes, while their sizes correspond to the number of individuals with a given haplotype. Small undescribed circles denote missing haplotypes. Names of the clades (see Figures S1 and S2) were assigned according to the naming proposed by Randi et al. (2004). Details on haplotypes in Table S1

Analysis of 3010 roe deer sequences of a fragment of the mtDNA control region (610 bp) revealed 328 haplotypes (Table 2) with 95 polymorphic sites (83 transitions, 18 transversions, and 4 indels). The numbers of samples representing each haplotype ranged from 1 to 177; 106 sequences were singletons (see Table S1). We identified 200 new haplotypes, not described in literature so far. The obtained sequences were deposited in GenBank under accession numbers ON368373–ON368700. Phylogenetic analyses revealed two major mtDNA lineages assigned to two species: the European roe deer *C*. *capreolus* (Cc) and the Siberian roe deer *C*. *pygargus* (Cp) (Figure 3; Figures S1 and S2). The majority of haplotypes (297 out of 328; 90.5%) grouped with the European roe deer lineage (Table 2). We detected 73 transitions (80.2%), 15 transversions (16.5%), and 3 indels (3.3%) in this group. Fifty‐seven mutation sites were polymorphic in Cc, but monomorphic in Cp. Roe deer exhibited high values of mtDNA diversity in both lineages: haplotype diversity (*H*_d_) was 0.982 in Cc and 0.831 in Cp, and nucleotide diversity (*π*) 0.11 and 0.10, respectively.

**TABLE 2:** Estimates of genetic diversity of mtDNA control region (610 bp) in the roe deer (*C*. *capreolus*) lineages, clades, and haplogroups (see Figure 3). Number of samples is indicated by *n* (numbers in parentheses correspond to samples collected and analyzed in this study), *h*—number of haplotypes, *S*—number of polymorphic sites, *H*_d_—haplotype diversity, π—nucleotide diversity, *k*—average number of pairwise differences. Haplotypes CcH53 and CcH55 (European lineage) were not assigned to any clade

Lineage, clade, haplogroup	*n*	*h*	*S*	*H*_d_	π	*k*
Siberian lineage	266 (257)	28	41	0.831	0.010	5.804
European lineage	2744 (1212)	300	84	0.982	0.011	6.401
Eastern clade	566 (375)	76	46	0.954	0.006	3.783
E1	77 (67)	6	5	0.174	0.000	0.180
E2	160 (60)	14	15	0.816	0.004	2.370
E3	140 (92)	22	16	0.900	0.004	2.243
E4	187 (154)	33	30	0.905	0.006	3.602
Central clade	2030 (795)	206	68	0.972	0.008	4.740
C1	737 (374)	83	42	0.951	0.006	3.817
C2	350 (207)	34	22	0.847	0.004	2.442
C3	124 (0)	5	4	0.124	0.000	0.127
C4	388 (139)	46	29	0.871	0.007	3.978
C5	12 (9)	3	6	0.439	0.002	1.379
C6	40 (33)	12	11	0.892	0.005	2.915
C7	328 (0)	10	11	0.639	0.002	1.341
C8	49 (31)	12	10	0.844	0.004	2.459
Western clade	148 (42)	18	21	0.901	0.008	4.802
W1	73 (36)	9	14	0.804	0.008	4.846
W2	75 (6)	9	11	0.798	0.004	2.708
CcH53	2 (2)	1	–	–	–	–
CcH55	2 (2)	1	–	–	–	–
Total	3010 (1469)	328	95	0.984	0.015	9.049

The European lineage showed a clear division into three major mtDNA clades (Figure 3; see Figures S1–S3). Following the names previously defined by Randi et al. (2004), we classified them as Eastern, Western, and Central clades. We distinguished a separate haplogroup for the roe deer subspecies *C*. *c*. *italicus* within the Central clade. All of them showed a wide range of haplotype diversity (largely proportional to the number of analyzed samples), and a relatively low nucleotide diversity (Table 2). The Central clade was most numerous: 74% of analyzed specimens and 67% of identified haplotypes in the European lineage belonged to it (Table 2). The Western clade, in turn, was least numerous, but it had the same level of nucleotide diversity as the Central one (*π* = 0.008). The Western clade had the highest average number of pairwise differences among all clades (Table 2), which suggests its nonmonophyletic origin and reflects the distribution of the clade and haplogroups on phylogenetic networks (Figure 3 and Figure S3). The Eastern clade had intermediate values in all estimates.

Further analysis of the haplotype genealogy of European roe deer lineage indicated the presence of an internal structuring in the described clades (Figure 3; Figure S3). Only haplotypes CcH55 (2 samples from Czech Republic) and CcH53 (1 sample from Slovakia and 1 from Serbia) were not assigned to any specific group. Inside the Eastern clade, we detected four haplogroups E1–E4 (Figure 3). The most numerous was haplogroup E4 (*n* = 187), while E1 was the least numerous (*n* = 77). Out of all the haplotypes that formed the haplogroup E1, only one (CcH220) was previously described (see Table S1).

The Central clade consisted of eight haplogroups (C1–C8), among which C1 had the highest number of individuals (*n* = 2030) and haplotypes (*h* = 206). The smallest recorded haplogroup was C5 (Table 2, Figure 3). Haplogroups C3 and C7 consisted of haplotypes found only in the published data. Those two haplogroups had the lowest average number of pairwise differences (0.127 and 1.341, respectively) and low nucleotide diversity (<0.001 and 0.002). All haplotypes grouped in haplogroup C7 were previously described in roe deer belonging to the subspecies *C*. *c*. *italicus* (comp. Randi et al., 2004).

The Western clade consisted of two haplogroups (W1 and W2) represented by similar numbers of individuals (Table 2). The haplogroup W1 had the highest average number of pairwise differences among all detected clades and haplogroups (*k* = 4.846) and the highest value of nucleotide diversity among all haplogroups (*π* = 0.008). The Western clade had many missing haplotypes (Figure 3 and Figure S3).

### 3.2 Spatial distribution of lineages, clades, and haplogroups

**FIGURE 4:**
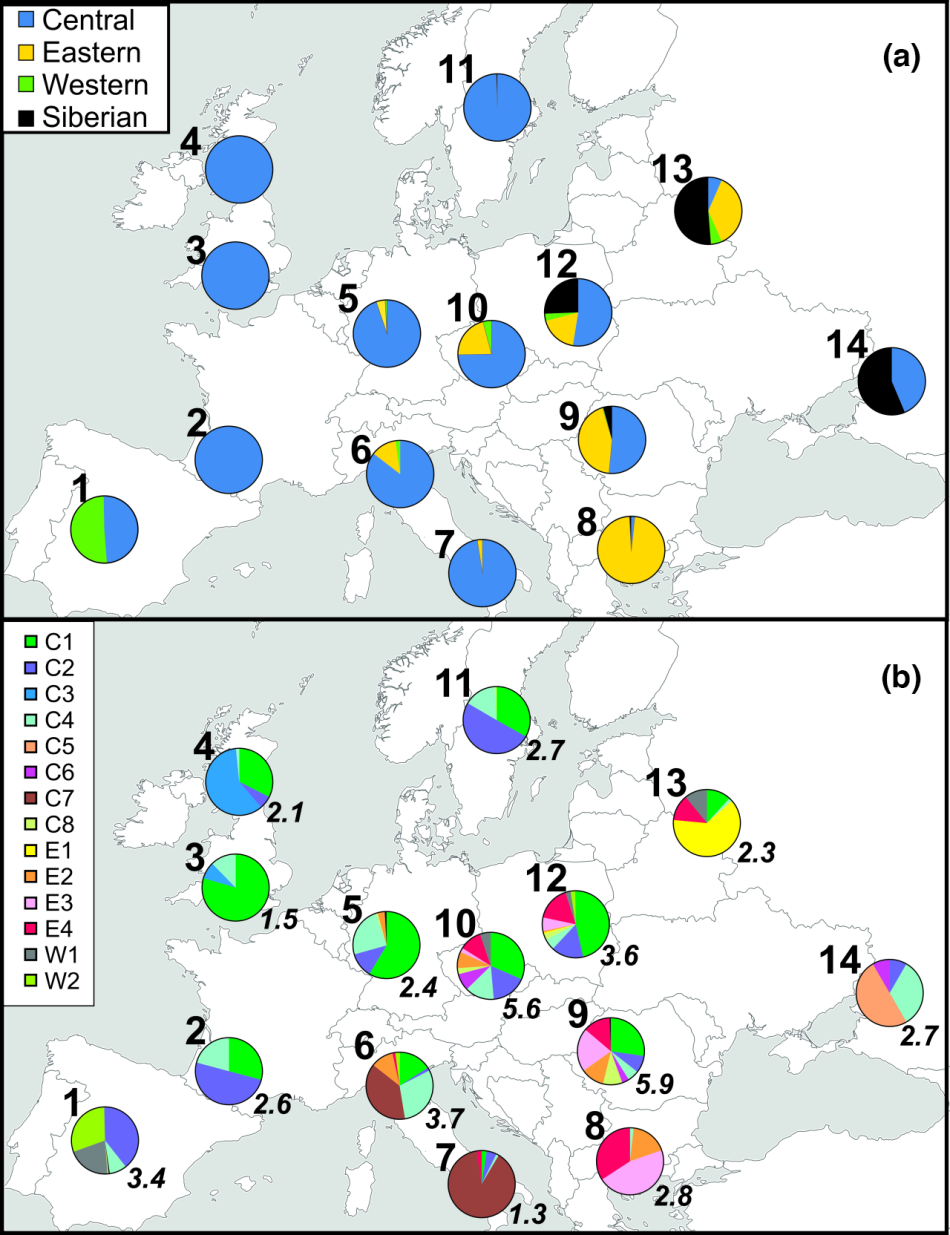
Haplotype frequencies of roe deer mtDNA groups in the defined 14 populations (see Figure 2) of European roe deer. Upper panel (a) depicts contributions of the Siberian lineage and the three main clades (Central, Eastern, and Western) in the European lineage in the 14 populations. Lower panel (b) shows the proportions of all haplogroups identified in the European lineage (see Figure 2). Numbers in italics are *B* indices indicating effective haplotype diversity

The spatial distribution of roe deer lineages in Europe showed a strong geographical pattern. Haplotypes of the Siberian lineage were recorded in western Russia, Finland, Estonia, Belarus, Lithuania, Poland, Ukraine, Slovakia, Romania, and Hungary (Figure S4). Their range fully overlapped with that of the European lineage. The highest proportion of the Siberian haplotypes was found in south‐eastern and eastern parts of roe deer geographical range (populations 14 and 13; 57 and 53% of samples, respectively), and it declined westwards (Figure 4a).

In the European lineage, we observed the spatial structuring of the three major clades (Figure S4). The Central clade was the most widespread and covered large part of the continent, with the highest frequencies in western, northern, and central regions (100% in populations 2–4 and 11, and 95% in population 5) (Figure 4a). This was the only roe deer clade which occurred in Great Britain and in the Scandinavian Peninsula. Some haplogroups of the Central clade were widely distributed (C1, C2, and especially C4), while others were geographically limited: C3—to Great Britain, C5—to the coastal parts of the Black Sea, C7—to the Apennine Peninsula (Figure 4b and Figure S5). The ranges of haplogroups C6 and C8 were restricted to east‐central Europe.

The Eastern clade was recorded in southern and east‐central Europe between 8° and 39°E longitude and below 56°N latitude (Figure S4). In Greece and Bulgaria, 99% of roe deer belonged to it, and the share of that clade declined northward (Figure 4a). The haplogroup E1 occurred only in Belarus, Lithuania, and the adjoining borderlands of Poland, Russia, and Ukraine (Figure 4b and Figure S6). Haplogroups E2–E4 considerably overlapped across the study area, yet some differences in their geographical ranges could still be observed (Figure 4b and Figure S6). For instance, individuals assigned to E3 were mainly located in the Balkan‐Dinaric and the Carpathian regions.

The Western clade was least numerous, but it was distributed over large areas of continental Europe from the Iberian Peninsula to western Russia (Figure S4 and Figure S7). None of the haplotypes from this clade was present north of 50°N. Except for Spain and Portugal (population 1), where it was recorded in 21% of roe deer samples, the Western clade contributed minimally (0.3–6%) to roe deer populations in central and eastern Europe (Figure 4a). Despite the low number of samples, inconsistency between the genetic position of the Western clade in roe deer phylogenies (Figure 3 and Figure S3), supported the division of this clade into two haplogroups (W1 and W2). Although haplogroups W1 and W2 showed substantial spatial overlap, their distributions were rather patchy (Figure 4b and Figure S4). Groups of related haplotypes in each of those haplogroups were endemic to some regions of Europe (Figure 4b and Figure S7).

We calculated the diversity index *B* of haplogroups belonging to the European lineage of roe deer in the 14 studied populations (Figure 4b). *B* index could vary from 1 to 14 (total number of haplogroups in the Eastern, Central, and Western clades). The highest diversity of phylogenetic haplogroups was found in the central part of roe deer range (populations 6, 9, 10, and 12; *B* from 3.7 to 5.9). All peripheral populations (except for population 1 in the Iberian Peninsula) showed low diversity (*B* from 1.3 to 2.8; Figure 4b).

### 3.3 Demographic history of roe deer

**FIGURE 5:**
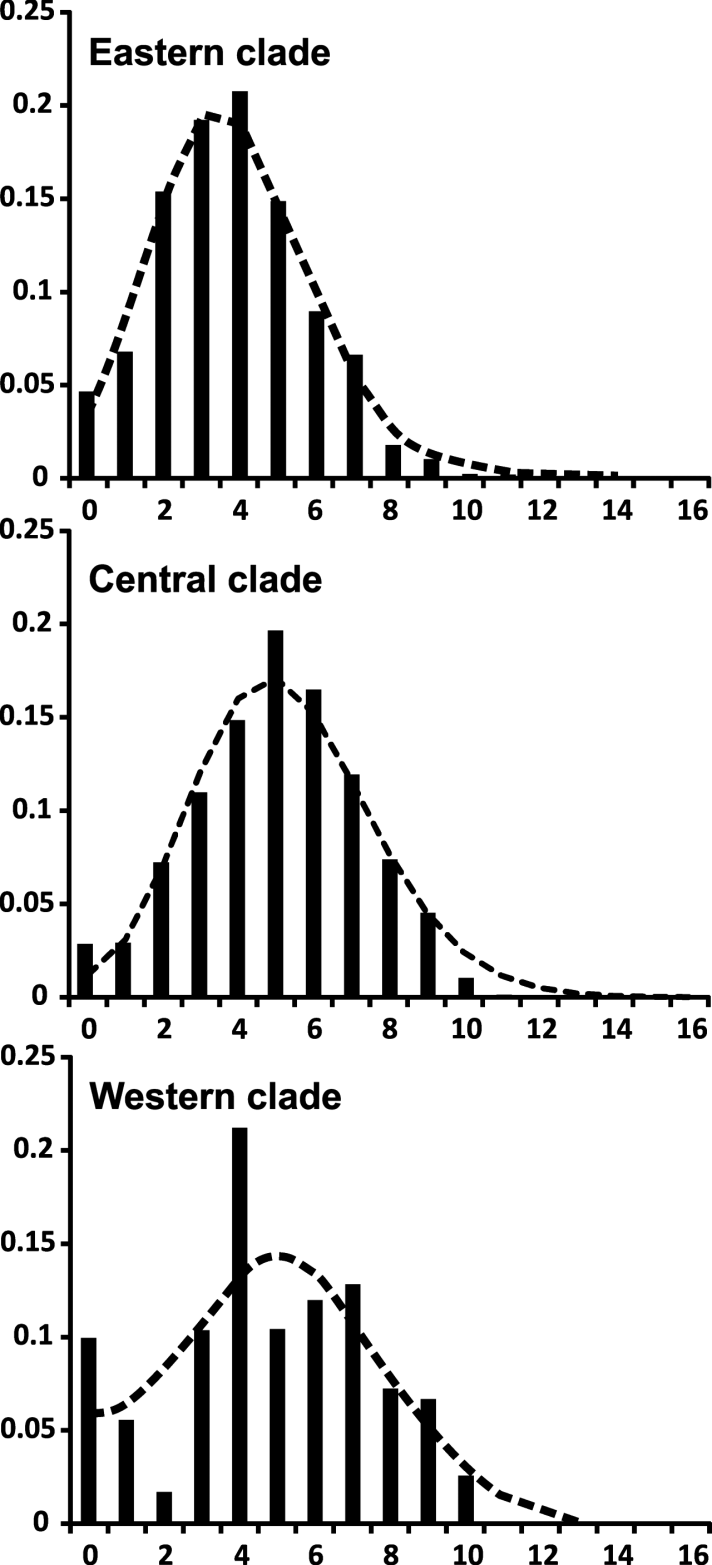
Mismatch distribution. Graphs represent the mismatch distribution for the three main European roe deer clades (Central, Eastern, and Western). Dashed lines correspond to the frequency expected based on the sudden expansion model

The goodness‐of‐fit tests, which compared expansion model with the observed mismatch distribution among individuals, showed evidence for expansion processes in all clades and nearly all haplogroups in the European lineage and in the Siberian lineage as well (Figure 5 and Figures S8 and S9). The observed distribution of pairwise differences did not deviate from the distribution expected under the expansion model. Additionally, values of Tajima's *D* and Fu's *F_S_* tests confirmed the recent expansion of haplogroups C3 and E1 (see Table S2). The only haplogroups not showing expansion tendency were C8 and W2, while C4 had only a weak support for expansion (see Table S2). The Central and Eastern clades exhibited strong unimodal distributions of the pairwise differences, which indicated one main expansion event in each case (Figure 5). The Western clade (Figure 5) and the Siberian lineage (see Figure S8) had multimodal distributions.

Expansion times were calculated for the spatial and demographic models based on two assumed mutation rates (see Methods). Therefore, the results*—*with very wide confidence intervals*—*can only give a broad view of the demographic history of European roe deer. Most of haplogroups of the Central and the Western clades showed expansion times predating the LGM (see Table S3). Possibly, only C2, C3, and C5 expanded during or after the LGM. The oldest expansion time, perhaps reaching as far as the Eemian interglacial (<100 ka BP), was found inhaplogroupW1. Three haplogroups in the Eastern clade (E1, E2, E3) showed the expansion time around or after the LGM (see Table S3).

## 4 DISCUSSION

We presented the comprehensive picture of the genetic diversity of roe deer*—*the most numerous, widespread, and ecologically flexible ungulate species of Europe. We identified the phylogeny within the species, mapped the spatial distribution of lineages, clades, and haplogroups, and attempted to describe their demographic history. By pooling together a large set of new data from central and eastern Europe and available literature data, we showed a clear, continent‐wide pattern of phylogeography of roe deer populations spanning from the Iberian Peninsula to western and south‐western Russia. However, there are still two important regions where data are lacking. The first is nearly the whole France (except for its southern part), and the second are the easternmost south‐eastern verges of roe deer range, including the Caucasus Mts., Turkey, and south‐eastern regions of the European part of Russia, where the range of *C*. *capreolus* overlaps with that of *C*. *pygargus*. Future research, filling those gaps in data coverage and analysis, cannot only broaden the pattern we presented in this work, but also verify some of our conclusions and interpretations.

The general picture of roe deer phylogeny and phylogeography we obtained in our study fully confirmed earlier findings (Lorenzini et al., 2014; Olano‐Marin et al., 2014; Randi et al., 2004; Royo et al., 2007; Sommer et al., 2009; Vernesi et al., 2002) showing two lineages (the European and the Siberian) of roe deer mtDNA in Europe, with the European lineage consisting of three clades (the Western, the Central, and the Eastern). Clear spatial phylogenetic patterns indicated, that translocations recorded in the historical times had rather minor impact and did not disturb the overall picture of the roe deer phylogeography in the continental scale. The major novelty of our study comes from the fact that we were able to divide the highly differentiated clades of the European lineage into well‐defined haplogroups, to map their ranges of occurrence, and quantify the spatial patterns of their frequencies in roe deer populations. Despite the sampling bias across the different populations and the inevitable inaccuracies in delimiting the phylogenetic haplogroups (marked as discrepancies in their branching pattern and haplotype membership in different phylogenetic trees and networks), the broad division of clades in the European lineage into smaller phylogenetic units appeared very informative for understanding the spatiotemporal history of the species.

### 4.1 The Siberian lineage of roe deer mtDNA

The introgression of mtDNA genes of the Siberian roe deer into *Capreolus capreolus* populations has already been detected in Hungary (Markov et al., 2016, 2017), Poland (Lorenzini et al., 2014; Matosiuk et al., 2014; Olano‐Marin et al., 2014), Lithuania (Lorenzini et al., 2014), Belarus (A. Danilkin, personal communication), Russia (Kashinina et al., 2018; Plakhina et al., 2014), and Ukraine (Danilkin et al., 2017; Kashinina et al., 2018). We found that the spatial range of the introgression is much wider than previously reported (it occurred also among roe deer in Finland, Estonia, Slovakia, and Romania), and the share of the Siberian mtDNA lineage in local populations declined from east to west. Despite a vast range of occurrence, the Siberian lineage was not very rich in haplotypes (28), yet it had a diversified phylogeny and most likely a complicated demographic history, which requires further studies.

The origin and sources of the introgression are not yet fully recognized. Some authors stated that it had been caused by an ancient hybridization during the postglacial range shifts of the European and the Siberian roe deer with subsequent range overlapping (Lorenzini et al., 2014; Matosiuk et al., 2014; Świsłocka et al., 2019). Conversely, Danilkin et al. (2017) and Kashinina et al. (2018) argued that the sources of hybridization were numerous relocations of the Siberian roe deer into the range of *C*. *capreolus* in the 19th and 20th centuries (Apollonio et al., 2014). Those two sources of introgression are most likely nonexclusive, as the recent human‐caused hybridization could have supplemented the ancient natural process. The problem can be elucidated by a future comparative study on the modern Siberian lineage of European roe deer and the modern and ancient phylogeny and phylogeography of the Siberian roe deer from their whole geographic range.

### 4.2 The European lineage of roe deer mtDNA*—*changes in time and space

The European roe deer was already present in Europe at least 600 ka BP and the large number of fossil and subfossil records evidenced that the species occurred in both glacial and interglacial phases since then (Sommer et al., 2009). Radiocarbon (C^14^) dating of roe deer fossil bones (a method that reaches only as far as 50–60 ka BP) showed that between 60 and 21 ka BP, roe deer occurred not only in the Mediterranean peninsulas, but also it repeatedly reached central Europe during milder interstadials (Sommer et al., 2009). Rapid climatic changes from colder to warmer periods were conducive to recurrent expansion–retreat cycles of roe deer and thus their genetic diversification. Indeed, most of haplogroups of the Central and Eastern clades and W2 of the Western clade could have originated in the Weichselian Pleniglacial, thus long before the Last Glacial Maximum.

**FIGURE 6:**
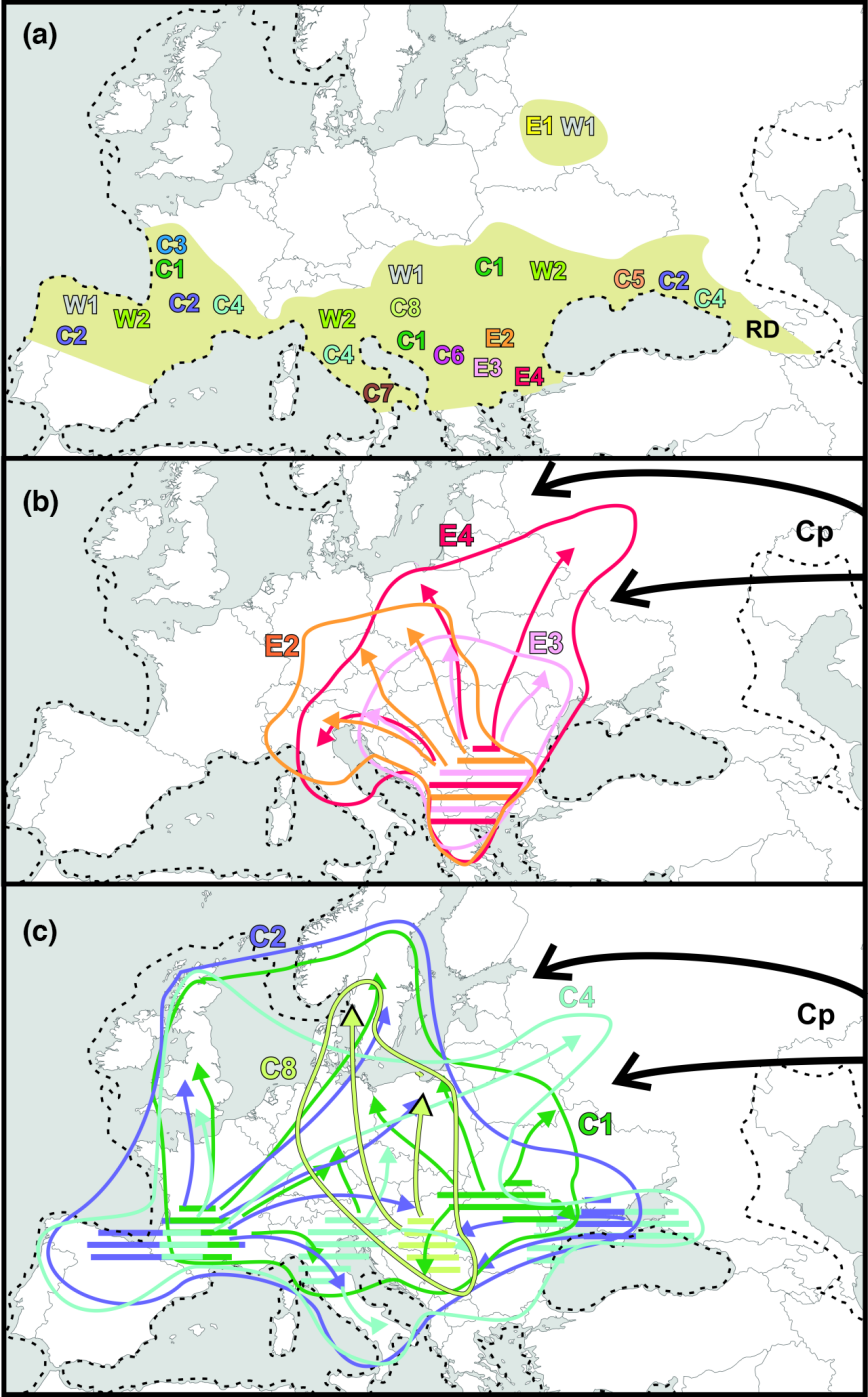
Reconstruction of the LGM refugial range of the European roe deer (a) and the most probable postglacial recolonization routes by the major haplogroups in the Eastern (b) and the Central clade (c). The LGM range reconstructed after Sommer et al. (2009), Stefaniak (2015), Markova and Puzachenko (2019), with modification over the Carpathians according to deciduous and coniferous tree range in the LGM after Tzedakis et al. (2013). Symbols of haplogroups as in Table 2 and Figure 3. RD—roe deer refugial population of unknown genetic profile. Cp—the Siberian lineage expansion into the European roe deer. Solid lines in (b) and (c) show the present ranges of occurrence of the haplogroups (see Figures S5 and S7)

Based on records of fossil and subfossil bones collated by Sommer et al. (2009), supplemented with data from Markova and Puzachenko (2019) for the eastern part of the continent, we reconstructed the LGM range of the European roe deer (Figure 6a). According to the synthesis of palynological data, Markova et al. (2008) proposed that roe deer dwelled in the Mediterranean and the Caucasian forests, and the southern variant of the periglacial forest‐steppe. Stefaniak (2015) reported the fossil bones of roe deer during the LGM from the Caucasus Mts. Thus, the species range during LGM spanned throughout southern Europe from the Iberian Peninsula and southern France to the eastern ranges of the Caucasus and embraced the southern part of the Carpathian region (Figure 6a). Further research on genetic diversity of roe deer in the Caucasus Mts. region is badly needed to elucidate the phylogeography of the species in the south‐eastern verges of its range. Below, we discuss the contemporary phylogeographic structure of roe deer and reconstruct the most likely phylogeography of the species during the LGM and its postglacial expansion.

The Central clade is by far the most widespread, numerous, and diversified among roe deer mtDNA clades. It occurs from Portugal, Spain, and Great Britain in western Europe to the Scandinavian Peninsula in the north, and the eastern shores of the Black Sea in the east. However, the Central clade is rare in the Balkan Peninsula, where its single, southernmost records were found in Bulgaria. Among eight haplogroups, C4 had the widest range (see Figure S5) and the oldest expansion time, however, with a wide confidence interval. Thus, we suggest that it might be a relic older than the Weichselian Glaciation. C4 might have then survived the LGM in southern France, northern Italy, and the north‐eastern shores of the Black Sea (Figure 6a), the areas indicated as suitable for the species during the LGM (Markova & Puzachenko, 2019). Its expansion could have started from those regions after the glacial period. Haplogroups C1 and C2 now are common in western and central Europe; we propose that the refugial areas of C1 were in southern France, the Dinaric Mts., and the eastern Carpathian region (Moldova), while those of C2*—*in the Iberian peninsula and southern France.

In addition to the earlier described Italian haplogroup, denoted as C7 in this paper, that occurred in the Apennine Peninsula only (Lorenzini et al., 2002; Mucci et al., 2012; Randi et al., 2004), we found three other haplogroups, each with a very restricted range: C5 recorded over the northern side of the Black Sea, C6 found mainly in Slovenia, Czech Republic and Slovakia (from Eastern Alps to Western Carpathians), and C3 found in Great Britain, only. The isolated haplogroups C5, C6, and C7 most likely did not expand from their LGM refugia (Figure 6a). Our haplogroup C3 is equivalent to the group, which supposed to represent the remnant Scottish population described by Baker and Hoelzel (2014) from northern Great Britain. The LGM refuge of the haplogroup C3 could have been located in present‐day France, near the land bridge existing in the past between northern France and southern England. During postglacial period, C3 roe deer colonized Great Britain, but—as proposed by Baker and Hoelzel (2014)*—*the subsequent human pressure led to the extinction of those ancient populations in the southern part of the British Isles and their replacement by introduced individuals. Today, the haplotypes belonging to haplogroup C3 from the primary postglacial colonization are still preserved in the northern populations (Baker & Hoelzel, 2014).

The distribution of the haplogroup C8 is similar to the localities of the southern spatially isolated haplogroups: the core of its range is located in the northwest of the Balkan Peninsula (in the Dinaric Mts.), which might have been its LGM refugium (Figure 6a).

The Eastern clade contained three haplogroups (E2–E4), which together constituted nearly 100% of roe deer samples in the south‐eastern Balkan Peninsula (the sampling region no 8: Bulgaria and Greece) and their share in local roe deer populations declined toward north. Obviously, the Balkan Peninsula was the glacial refuge of haplogroups E2, E3, and E4. The situation of an isolated haplogroup E1 is different. It now occurs largely in regions that had been ice covered during the LGM (Lithuania, Belarus, western Russia), and its spatial expansion times suggested recent immigration. E1 consists of basically one haplotype with its few derivatives differing by one mutation step. We suggest that it survived the LGM in a northern refuge located at high latitude on the Belarussian‐Russian borderland (Figure 6a), where boreal coniferous trees were documented to grow during the LGM (Svenning et al., 2008; Tzedakis et al., 2013). Heikkilä et al. (2009) documented the presence of *Betula*, *Pinus*, and *Picea* populations in the eastern Baltic region (Latvia) already in the Late Glacial. This suggested that the full‐glacial locations of those trees were in the regions south and south‐east of Latvia, possibly at 55–60°N, making the area habitable even for large mammals. Interestingly, the pan‐European study on molecular biogeography of wild boar *S. scrofa* (Niedziałkowska, Tarnowska, et al., 2021) discovered a rare, ancient clade of the species in the Russian part of the range of roe deer haplogroup E1. Also, the Europe‐wide study of wolf *Canis lupus* population by SNP analysis (Stronen et al., 2013) found that the western and northern Belarus wolves showed the most divergent genotypes within north‐central Europe.

The Western clade, discovered for the first time in the western part of the continent, appeared “Western” no longer, as we found it also in central and eastern Europe, where it formed up to 5.3% of roe deer in the sampling region no 13 (Belarus, Lithuania, and western Russia). We decided to retain the name “Western” for this group, as it has already been well established in literature. The internal phylogeny of the Western clade (many missing, possibly extinct haplotypes), discontinuity of its occurrence over the continent, spatial segregation of groups of haplotypes, and very old expansion times strongly suggest that the Western clade has been a relic of the Eemian interglacial (MIS 5) 130–80 ka BP, when it probably had occurred throughout the continent. This could also explain why results from different reconstructions (see Figure 3 and Figure S3) do not seem coherent. Many missing haplotypes could have blurred the position of the Western clade in the phylogeographic networks. The present occurrence and diversification of the Western clade suggest at least three glacial refugia: the Iberian Peninsula, southern Alps, and the Carpathian region *sensu lato*. In addition, the haplogroup W1 had survived the LGM in the north‐eastern refuge located in the present‐day Belarussian‐Russian borderland (Figure 6a).

### 4.3 Postglacial colonization of Europe by roe deer

Our data suggest that different haplogroups of roe deer contributed differently to the postglacial colonization of the European continent. Among 14 haplogroups belonging to three mtDNA clades, seven played the major roles in recolonization of the postglacial habitats. These were, from the most successful: C2, C1, C4, E4, E2, E3, and C8 (Figure 6a,b). The other seven haplogroups showed rather weak expansion or most probably remained in their LGM refugia.

Also, different regions of the large, glacial refugial area could have contributed unevenly to the modern range of roe deer. The southern France refugial population might have expanded to cover the whole western and north‐western Europe, and possibly reached also central and eastern parts of the continent (Figure 6c). Then, the Balkan refugial area, especially its eastern part, gave rise to the vast range of central and eastern Europe being repopulated by the Eastern clade of roe deer (Figure 6b), whereas colonizers from the Dinaric Mts. region (mainly C8) dispersed northward in a fairly narrow belt, probably between the western and eastern expanding populations.

The glacial populations of roe deer from the southernmost and easternmost refugial regions (the Iberian and the Apennine Peninsulas, the eastern Carpathians and the north‐eastern Black Sea shore) did not seem to expand much in the postglacial period. While the Iberian and Apennine roe deer were located behind the expanding populations, the expansion from the eastern Carpathians and the Black Sea region might have been halted by the Siberian lineage of roe deer expanding from the East (Figure 6b,c). The study focused on the Siberian lineage is needed to understand the mechanisms of that expansion and its influence on the genetic profile of European roe deer populations in the eastern part of the species range.

## 5 CONCLUSIONS

Our data suggest that the present phylogeographic pattern of roe deer in Europe might have been shaped by the Weichselian pleniglacial diversification of haplogroups, survival of the Eemian relic clade and other clades in multiple refugial areas, the postglacial recolonization, and hybridization with the Siberian roe deer. Modern relocations and exploitation by humans probably did not significantly affect the observed phylogeographical patterns and had an impact only in a local scale. The proposed recolonization patterns and hybridization with *C*. *pygargus* need further genetic studies, including also ancient samples of the roe deer, to reveal with more details the evolutionary history of those two sister species in Eurasia.

## AUTHOR CONTRIBUTIONS

**Kamila Plis:** Data curation (lead); formal analysis (lead); visualization (equal); writing – original draft (lead). **Magdalena Niedziałkowska:** Conceptualization (equal); formal analysis (supporting); methodology (supporting); writing – original draft (supporting); writing – review and editing (lead). **Tomasz Borowik:** Data curation (supporting); writing – original draft (supporting). **Johannes Lang:** Data curation (supporting); Writing – original draft (supporting); writing – review and editing (supporting). **Mike Heddergott:** Data curation (equal); Writing – original draft (supporting); writing – review and editing (equal). **Juha Tiainen:** Data curation (equal); writing – original draft (supporting); writing – review and editing (equal). **Aleksey Bunevich:** Data curation (equal). **Nikica Šprem:** Data curation (equal); writing – original draft (supporting); writing – review and editing (equal). **Ladislav Paule:** Data curation (equal); Supervision (equal); writing – original draft (equal); writing – review and editing (equal). **Aleksey Danilkin:** Conceptualization (supporting); data curation (lead); writing – original draft (equal); writing – review and editing (equal). **Marina Kholodova:** Data curation (supporting); Writing – review and editing (equal). **Elena Zvychaynaya:** Data curation (supporting); writing – review and editing (supporting). **Nadezhda Kashinina:** Data curation (supporting); writing – review and editing (supporting). **Boštjan Pokorny:** Data curation (equal); writing – original draft (supporting); writing – review and editing (equal). **Katarina Flajšman:** Data curation (equal); writing – original draft (supporting); writing – review and editing (equal). **Algimantas Paulauskas:** Conceptualization (supporting); data curation (equal); writing – original draft (supporting); writing – review and editing (equal). **Mihajla Djan:** Data curation (equal); writing – original draft (supporting); writing – review and editing (supporting). **Zoran Ristić:** Data curation (equal); writing – review and editing (supporting). **Luboš Novák:** Data curation (equal); writing – review and editing (supporting). **Szilvia Kusza:** Data curation (equal); writing – original draft (supporting); writing – review and editing (supporting). **Christine Miller:** Data curation (equal); writing – original draft (supporting); writing – review and editing (supporting). **Dimitris Tsaparis:** Data curation (supporting); writing – original draft (equal); writing – review and editing (equal). **Stoyan Stoyanov:** Data curation (equal); writing – original draft (equal); writing – review and editing (equal). **Maryna Shkvyria:** Data curation (equal); writing – original draft (equal); writing – review and editing (equal). **Franz Suchentrunk:** Conceptualization (supporting); data curation (equal); writing – original draft (equal); writing – review and editing (equal). **Miroslav Kutal:** Data curation (equal); writing – original draft (supporting); writing – review and editing (supporting). **Vukan Lavadinović:** Data curation (supporting); writing – review and editing (supporting). **Dragana Šnjegota:** Data curation (equal); formal analysis (supporting); writing – original draft (equal); writing – review and editing (equal). **Ana‐Maria Krapal:** Data curation (supporting); investigation (supporting); writing – original draft (supporting); writing – review and editing (supporting). **Gabriel Dănilă:** Data curation (supporting); writing – original draft (supporting); writing – review and editing (supporting). **Rauno Veeroja:** Data curation (supporting); writing – original draft (supporting); writing – review and editing (supporting). **Elżbieta Dulko:** Data curation (supporting); formal analysis (equal); writing – original draft (supporting); writing – review and editing (supporting). **Bogumiła Jędrzejewska:** Conceptualization (lead); funding acquisition (lead); investigation (equal); supervision (lead); visualization (equal); writing – original draft (lead); writing – review and editing (lead).

## CONFLICT OF INTEREST

The authors declare that they have no conflict of interest.

## Supporting information

Table S1

Table S2

Table S3

Figures S1‐S9

## Data Availability

The authors confirm that all data underlying the findings are fully available without restriction. The data has been submitted to dryad with the Data Identifier https://doi.org/10.5061/dryad.76hdr7t01. Complete mtDNA sequences have been deposited in GenBank with accession numbers ON368373–ON368700.
