# Supplementary material for: Pan‐European phylogeography of the European roe deer (Capreolus capreolus)
Source: Ecol Evol. 2022 May 19;12(5):e8931. doi: 10.1002/ece3.8931 (PMC9120558; doi:10.1002/ece3.8931)
Supplement: Supplementary file 1 — Table S1 [file ECE3-12-e8931-s004.docx]

Table S1. The list of haplotypes and their frequency of occurrence in analysed data set with corresponding names from the reference data. Haplotypes, which were presented as singletons in our own data, were marked with stars and red color (e.g. *CcH9.) Division into clades followed classification proposed by Randi et al. (2004). For the group compositions see Figure 2. Some of sequences indicated in literature as a separate haplotypes, were pooled to one due to trimming to common length 610 bp.

| Clade | Group | Haplotype | Number of samples | |  |  |  |  |  |  |  |
| --- | --- | --- | --- | --- | --- | --- | --- | --- | --- | --- | --- |
|  |  |  | This study | Literature | Randi 2004 | Gentile 2009 | Baker 2013 | Lorenzini 2014 | Biosa 2015 |  |  |
| **Central** | **C1** | CcH1 | 3 |  |  |  |  |  |  |  |  |
|  |  | CcH4 | 2 |  |  |  |  |  |  |  |  |
|  |  | CcH8 | 2 | 2 | H37 |  |  |  |  |  |  |
|  |  | *CcH9 | 1 |  |  |  |  |  |  |  |  |
|  |  | CcH11 | 2 |  |  |  |  |  |  |  |  |
|  |  | *CcH14 | 1 |  |  |  |  |  |  |  |  |
|  |  | CcH20 | 5 |  |  |  |  |  |  |  |  |
|  |  | *CcH21 | 1 |  |  |  |  |  |  |  |  |
|  |  | *CcH22 | 1 |  |  |  |  |  |  |  |  |
|  |  | CcH25 | 5 |  |  |  |  |  |  |  |  |
|  |  | CcH26 | 52 | 19 | H48 | L17 | h10, h12 | Cc32 | B01, B03 |  |  |
|  |  | CcH27 | 2 |  |  |  |  |  |  |  |  |
|  |  | CcH28 | 2 |  |  |  |  |  |  |  |  |
|  |  | *CcH29 | 1 |  |  |  |  |  |  |  |  |
|  |  | CcH30 | 2 |  |  |  |  |  |  |  |  |
|  |  | CcH31 | 4 |  |  |  |  |  |  |  |  |
|  |  | CcH33 | 2 |  |  |  |  |  |  |  |  |
|  |  | CcH41 | 10 | 4 | H47 |  | h27 | Cc23 |  |  |  |
|  |  | CcH42 | 2 |  |  |  |  |  |  |  |  |
|  |  | CcH57 | 5 |  |  |  |  |  |  |  |  |
|  |  | *CcH58 | 1 |  |  |  |  |  |  |  |  |
|  |  | *CcH61 | 1 |  |  |  |  |  |  |  |  |
|  |  | CcH66 | 25 | 22 | H41 | L14, L15 |  |  |  |  |  |
|  |  | CcH72 | 5 | 2 | H67 |  |  |  |  |  |  |
|  |  | CcH73 | 3 |  | H133 |  |  |  |  |  |  |
|  |  | *CcH75 | 1 |  |  |  |  |  |  |  |  |
|  |  | CcH78 | 2 |  |  |  |  |  |  |  |  |
|  |  | *CcH79 | 1 | 59 | H24 | L18 | h03 | Cc18 |  |  |  |
|  |  | *CcH80 | 1 |  |  |  |  |  |  |  |  |
|  |  | CcH81 | 3 |  |  |  |  |  |  |  |  |
|  |  | *CcH86 | 1 |  |  |  |  |  |  |  |  |
|  |  | *CcH88 | 1 |  |  |  |  |  |  |  |  |
|  |  | CcH96 | 20 | 3 | H138 |  |  |  |  |  |  |
|  |  | CcH104 | 7 |  |  |  |  |  |  |  |  |
|  |  | *CcH116 | 1 |  |  |  |  |  |  |  |  |
|  |  | *CcH117 | 1 |  |  |  |  |  |  |  |  |
|  |  | *CcH119 | 1 |  |  |  |  |  |  |  |  |
|  |  | CcH128 | 4 |  |  |  |  |  |  |  |  |
|  |  | *CcH131 | 1 |  |  |  |  |  |  |  |  |
|  |  | *CcH134 | 1 |  |  |  |  |  |  |  |  |
|  |  | CcH135 | 3 | 49 |  |  | h02 |  |  |  |  |
|  |  | CcH136 | 2 |  |  |  |  |  |  |  |  |
|  |  | *CcH140 | 1 |  |  |  |  |  |  |  |  |
|  |  | CcH141 | 2 | 3 | H98 |  |  | Cc12 |  |  |  |
|  |  | *CcH149 | 1 |  |  |  |  |  |  |  |  |
|  |  | CcH163 | 2 | 5 | H63 |  |  |  |  |  |  |
|  |  | CcH164 | 59 | 3 | H38 |  |  |  |  |  |  |
|  |  | CcH165 | 4 |  |  |  |  |  |  |  |  |
|  |  | CcH168 | 35 | 3 | H54 |  |  |  |  |  |  |
|  |  | CcH169 | 7 |  |  |  |  |  |  |  |  |
|  |  | CcH170 | 8 |  |  |  |  |  |  |  |  |
|  |  | CcH172 | 3 |  |  |  |  |  |  |  |  |
|  |  | CcH173 | 8 |  |  |  |  |  |  |  |  |
|  |  | CcH177 | 5 | 19 | H84 |  |  |  |  |  |  |
|  |  | CcH179 | 7 |  |  |  |  |  |  |  |  |
|  |  | CcH181 | 3 | 4 | H107 |  |  |  |  |  |  |
|  |  | CcH187 | 2 | 67 | H14 | L7 |  |  |  |  |  |
|  |  | CcH190 | 8 |  |  |  |  |  |  |  |  |
|  |  | *CcH193 | 1 |  |  |  |  |  |  |  |  |
|  |  | CcH195 | 6 |  |  |  |  |  |  |  |  |
|  |  | CcH199 | 3 | 1 |  |  |  | Cc19 |  |  |  |
|  |  | CcH202 | 2 | 0 |  |  |  |  |  |  |  |
|  |  | *CcH208 | 1 |  |  |  |  |  |  |  |  |
| Table S1 - continued | | | | | | | | | |  |  |
| **Central** | **C1** | CcH210 | 2 |  |  |  |  |  |  |  |  |
|  |  | *CcH214 | 1 |  |  |  |  |  |  |  |  |
|  |  | CcH215 | 2 |  |  |  |  |  |  |  |  |
|  |  | CcH216 | 3 | 9 |  |  | h13 |  |  |  |  |
|  |  | *CcH222 | 1 |  |  |  |  |  |  |  |  |
|  |  | *CcH226 | 1 |  |  |  |  |  |  |  |  |
|  |  | CcH227 | 3 |  |  |  |  |  |  |  |  |
|  |  | *CcH229 | 1 |  |  |  |  |  |  |  |  |
|  |  | *CcH231 | 1 |  |  |  |  |  |  |  |  |
|  |  | BAH07 |  | 35 | H148 |  | h07, h22 |  |  |  |  |
|  |  | BAH08 |  | 7 |  |  | h08 |  |  |  |  |
|  |  | BAH15 |  | 2 |  |  | h15 |  |  |  |  |
|  |  | BAH19 |  | 1 |  |  | h19 |  |  |  |  |
|  |  | RANH122 |  | 2 | H122 |  |  |  |  |  |  |
|  |  | RANH117 |  | 6 | H117 |  |  |  |  |  |  |
|  |  | RANH103 |  | 13 | H103 |  |  |  |  |  |  |
|  |  | RANH97 |  | 2 | H97 |  |  |  |  |  |  |
|  |  | RANH85 |  | 6 | H85 |  |  |  |  |  |  |
|  |  | RANH62 |  | 6 | H62 |  |  | Cc22 |  |  |  |
|  |  | RANH22 |  | 2 | H22 |  |  |  |  |  |  |
| **Central** | **C2** | *CcH17 | 1 |  |  |  |  |  |  |  |  |
|  |  | *CcH48 | 1 | 10 |  |  |  |  |  |  |  |
|  |  | *CcH68 | 1 |  |  |  |  |  |  |  |  |
|  |  | *CcH125 | 1 |  |  |  |  |  |  |  |  |
|  |  | CcH133 | 7 |  |  |  |  |  |  |  |  |
|  |  | CcH139 | 2 |  |  |  |  |  |  |  |  |
|  |  | *CcH150 | 1 |  |  |  |  |  |  |  |  |
|  |  | *CcH154 | 1 |  |  |  |  |  |  |  |  |
|  |  | *CcH158 | 1 |  | H51 |  |  |  |  |  |  |
|  |  | CcH160 | 3 |  |  |  |  |  |  |  |  |
|  |  | CcH167 | 18 | 9 | H83 |  |  | Cc25 |  |  |  |
|  |  | CcH171 | 8 |  |  |  |  |  |  |  |  |
|  |  | CcH178 | 2 |  |  |  |  |  |  |  |  |
|  |  | *CcH180 | 1 | 3 | H89 |  |  |  |  |  |  |
|  |  | CcH189 | 4 |  |  |  |  |  |  |  |  |
|  |  | *CcH191 | 1 |  |  |  |  |  |  |  |  |
|  |  | CcH196 | 98 | 19 | H21 |  | h21 | Cc24 |  |  |  |
|  |  | *CcH200 | 1 |  |  |  |  |  |  |  |  |
|  |  | CcH201 | 27 | 9 | H137 |  | h01 |  |  |  |  |
|  |  | CcH211 | 18 |  | H66 |  |  |  |  |  |  |
|  |  | CcH219 | 9 | 23 | H158 |  | h11 | Cc14 |  |  |  |
|  |  | *CcH228 | 1 |  |  |  |  |  |  |  |  |
|  |  | BAH06 |  | 4 |  |  | h06 |  |  |  |  |
|  |  | BAH16 |  | 2 |  |  | h16 |  |  |  |  |
|  |  | BAH17 |  | 2 |  |  | h17 |  |  |  |  |
|  |  | BAH20 |  | 1 |  |  | h20 |  |  |  |  |
|  |  | RANH159 |  | 14 | H159 |  |  | Cc13 |  |  |  |
|  |  | RANH74 |  | 2 | H74 |  |  |  |  |  |  |
|  |  | RANH2 |  | 4 | H2 |  |  |  |  |  |  |
|  |  | RANH1 |  | 43 | H1, H5 |  |  | Cc21 |  |  |  |
|  |  | LORCC20 |  | 1 |  |  |  | Cc20 |  |  |  |
|  |  | GENL8 |  | 2 |  | L8 |  |  |  |  |  |
|  |  | GENL9 |  | 1 |  | L9 |  |  |  |  |  |
|  |  | GENL10 |  | 1 |  | L10 |  |  |  |  |  |
| **Central** | **C3** | BAH05 |  | 116 |  |  | h05, h23 |  |  |  |  |
|  |  | BAH09 |  | 2 |  |  | h09 |  |  |  |  |
|  |  | BAH14 |  | 4 |  |  | h14, h25 |  |  |  |  |
|  |  | BAH24 |  | 1 |  |  | h24 |  |  |  |  |
|  |  | BAH26 |  | 1 |  |  | h26 |  |  |  |  |
| **Central** | **C4** | CcH7 | 11 | 35 | H18 |  |  |  | B02, B09, B11 |  |  |
|  |  | CcH10 | 8 | 19 | H12 | L12 |  |  |  |  |  |
|  |  | *CcH18 | 1 | 15 | H52 |  |  |  | B07 |  |  |
|  |  | CcH19 | 4 | 118 | H13 | L11 |  | Cc17 | B04 |  |  |
|  |  | CcH39 | 6 |  |  |  |  |  |  |  |  |
|  |  | CcH43 | 3 |  |  |  |  |  |  |  |  |
|  |  | *CcH44 | 1 |  |  |  |  |  |  |  |  |
|  |  | *CcH69 | 1 |  |  |  |  |  |  |  |  |
|  |  | CcH70 | 2 |  |  |  |  |  |  |  |  |
|  |  | CcH71 | 7 | 17 | H46 |  | h04 |  |  |  |  |
|  |  | CcH77 | 4 |  |  |  |  |  |  |  |  |
|  |  | CcH82 | 2 |  |  |  |  |  |  |  |  |
|  |  | *CcH83 | 1 |  |  |  |  |  |  |  |  |
| Table S1 - continued | | | | | | | | | |  |  |
| **Central** | **C4** | *CcH87 | 1 |  |  |  |  |  |  |  |  |
|  |  | *CcH105 | 1 |  |  |  |  |  |  |  |  |
|  |  | CcH108 | 4 | 9 | H80 |  |  |  |  |  |  |
|  |  | *CcH122 | 1 |  |  |  |  |  |  |  |  |
|  |  | *CcH124 | 1 |  |  |  |  |  |  |  |  |
|  |  | CcH126 | 4 | 2 |  |  |  |  | B06 |  |  |
|  |  | CcH127 | 6 |  |  |  |  |  |  |  |  |
|  |  | *CcH130 | 1 |  |  |  |  |  |  |  |  |
|  |  | *CcH132 | 1 |  |  |  |  |  |  |  |  |
|  |  | CcH137 | 6 |  |  |  |  |  |  |  |  |
|  |  | CcH143 | 2 | 4 | H31 |  |  | Cc26 |  |  |  |
|  |  | *CcH145 | 1 |  |  |  |  |  |  |  |  |
|  |  | CcH146 | 2 |  |  |  |  |  |  |  |  |
|  |  | *CcH157 | 1 |  |  |  |  |  |  |  |  |
|  |  | CcH161 | 21 |  |  |  |  |  |  |  |  |
|  |  | CcH183 | 6 |  |  |  |  |  |  |  |  |
|  |  | CcH192 | 7 |  |  |  |  |  |  |  |  |
|  |  | CcH194 | 6 |  |  |  |  |  |  |  |  |
|  |  | CcH204 | 2 |  |  |  |  |  |  |  |  |
|  |  | CcH205 | 8 |  |  |  |  |  |  |  |  |
|  |  | CcH223 | 2 |  |  |  |  |  |  |  |  |
|  |  | CcH224 | 4 |  |  |  |  |  |  |  |  |
|  |  | BAH18 |  | 1 |  |  | h18 |  |  |  |  |
|  |  | BIB10 |  | 1 |  |  |  |  | B10 |  |  |
|  |  | BIB08 |  | 1 |  |  |  |  |  |  |  |
|  |  | BIB05 |  | 1 |  |  |  |  | B05 |  |  |
|  |  | RANH95 |  | 2 | H95 |  |  |  |  |  |  |
|  |  | RANH68 |  | 1 | H68 |  |  |  |  |  |  |
|  |  | RANH4 |  | 2 | H4 |  |  |  |  |  |  |
|  |  | RANH3 |  | 9 | H3, H78 |  |  | Cc11 |  |  |  |
|  |  | LORCC10 |  | 4 |  | L16 |  | Cc10 |  |  |  |
|  |  | LORCC7 |  | 7 |  |  |  | Cc7 |  |  |  |
|  |  | GENL13 |  | 1 |  | L13 |  |  |  |  |  |
| **Central** | **C5** | *CcH35 | 1 | 1 |  |  |  | Cc15 |  |  |  |
|  |  | *CcH36 | 1 |  |  |  |  |  |  |  |  |
|  |  | CcH40 | 7 | 2 |  |  |  | Cc16 |  |  |  |
| **Central** | **C6** | CcH12 | 8 |  |  |  |  |  |  |  |  |
|  |  | *CcH13 | 1 | 2 | H87, H143 |  |  |  |  |  |  |
|  |  | *CcH23 | 1 |  |  |  |  |  |  |  |  |
|  |  | *CcH24 | 1 |  |  |  |  |  |  |  |  |
|  |  | CcH54 | 8 |  |  |  |  |  |  |  |  |
|  |  | CcH56 | 2 |  |  |  |  |  |  |  |  |
|  |  | CcH59 | 5 | 1 |  |  |  | Cc31 |  |  |  |
|  |  | CcH74 | 4 |  |  |  |  |  |  |  |  |
|  |  | *CcH144 | 1 |  |  |  |  |  |  |  |  |
|  |  | CcH186 | 2 |  |  |  |  |  |  |  |  |
|  |  | RANH145 |  | 2 | H145 |  |  |  |  |  |  |
|  |  | RANH141 |  | 2 | H141 |  |  |  |  |  |  |
| **Central** | **C7** | BIB13 |  | 177 | H15 | L5 |  |  | B13, B15, B16, B19, B20, B21, B22, B23, |  |  |
|  |  | BIB18 |  | 1 |  |  |  |  | B18 |  |  |
|  |  | BIB17 |  | 26 | H43 | L4 |  |  | B17 |  |  |
|  |  | BIB14 |  | 1 |  |  |  |  | B14 |  |  |
|  |  | RANH60 |  | 15 | H60 | L6 |  | Cc30 |  |  |  |
|  |  | RANH45 |  | 2 | H45 |  |  |  |  |  |  |
|  |  | RANH42 |  | 2 | H42 |  |  |  |  |  |  |
|  |  | RANH40 |  | 24 | H40 |  |  | Cc29 |  |  |  |
|  |  | RANH35 |  | 79 | H35, H36 | L2, L3 |  | Cc27, Cc28 |  |  |  |
|  |  | GENL1 |  | 1 |  | L1 |  |  |  |  |  |
| **Central** | **C8** | *CcH3 | 1 |  |  |  |  |  |  |  |  |
|  |  | CcH5 | 4 |  | H55 |  |  |  |  |  |  |
|  |  | *CcH34 | 1 |  |  |  |  |  |  |  |  |
|  |  | *CcH45 | 1 |  |  |  |  |  |  |  |  |
|  |  | *CcH60 | 1 |  |  |  |  |  |  |  |  |
|  |  | CcH64 | 10 | 3 | H104 |  |  |  |  |  |  |
|  |  | CcH176 | 3 | 10 | H86 |  |  |  |  |  |  |
|  |  | CcH209 | 3 |  |  |  |  |  |  |  |  |
|  |  | *CcH213 | 1 |  |  |  |  |  |  |  |  |
|  |  | CcH230 | 6 |  |  |  |  |  |  |  |  |
|  |  | RANH118 |  | 2 | H118 |  |  |  |  |  |  |
|  |  | RANH7 |  | 3 | H7 |  |  |  |  |  |  |
| **Central** | **CcH55** | CcH55 | 2 |  |  |  |  |  |  |  |  |
| **East** | **CcH53** | CcH53 | 2 |  |  |  |  |  |  |  |  |
| Table S1 - continued | | | | | | | | | |  |  |
| **East** | **E1** | *CcH38 | 1 |  |  |  |  |  |  |  |  |
|  |  | CcH47 | 3 |  |  |  |  |  |  |  |  |
|  |  | *CcH62 | 1 |  |  |  |  |  |  |  |  |
|  |  | *CcH63 | 1 |  |  |  |  |  |  |  |  |
|  |  | *CcH67 | 1 |  |  |  |  |  |  |  |  |
|  |  | CcH220 | 60 | 10 |  |  |  | Cc34 |  |  |  |
| **East** | **E2** | CcH6 | 13 | 33 | H16 | L19 |  | Cc8 | B12 |  |  |
|  |  | *CcH32 | 1 |  |  |  |  |  |  |  |  |
|  |  | CcH89 | 8 | 7 | H29 | L21 |  |  |  |  |  |
|  |  | *CcH111 | 1 |  |  |  |  |  |  |  |  |
|  |  | CcH118 | 8 |  |  |  |  |  |  |  |  |
|  |  | CcH120 | 2 | 2 | H10 |  |  |  |  |  |  |
|  |  | *CcH129 | 1 |  |  |  |  |  |  |  |  |
|  |  | CcH153 | 2 | 34 | H27 | L23 |  |  |  |  |  |
|  |  | CcH174 | 2 |  |  |  |  |  |  |  |  |
|  |  | CcH188 | 20 | 12 | H23 | L20 |  | Cc9 |  |  |  |
|  |  | *CcH207 | 1 |  |  |  |  |  |  |  |  |
|  |  | *CcH225 | 1 |  |  |  |  |  |  |  |  |
|  |  | RANH121 |  | 4 | H121 |  |  |  |  |  |  |
|  |  | RANH111 |  | 8 | H111 |  |  |  |  |  |  |
| **East** | **E3** | CcH49 | 17 | 6 | H102, H17 |  |  |  |  |  |  |
|  |  | CcH65 | 4 |  | H140, H150 |  |  |  |  |  |  |
|  |  | CcH91 | 3 | 6 | H82 |  |  |  |  |  |  |
|  |  | *CcH94 | 1 | 3 | H90 |  |  |  |  |  |  |
|  |  | *CcH95 | 1 |  |  |  |  |  |  |  |  |
|  |  | *CcH98 | 1 |  |  |  |  |  |  |  |  |
|  |  | CcH99 | 2 |  |  |  |  |  |  |  |  |
|  |  | CcH101 | 3 |  |  |  |  |  |  |  |  |
|  |  | CcH103 | 3 | 2 | H109 |  |  |  |  |  |  |
|  |  | CcH106 | 4 | 2 |  |  |  | Cc35 |  |  |  |
|  |  | CcH109 | 2 | 12 | H11 |  |  |  |  |  |  |
|  |  | *CcH112 | 1 |  |  |  |  |  |  |  |  |
|  |  | CcH113 | 3 |  |  |  |  |  |  |  |  |
|  |  | *CcH114 | 1 |  |  |  |  |  |  |  |  |
|  |  | *CcH148 | 1 | 4 | H120 |  |  |  |  |  |  |
|  |  | CcH152 | 2 | 2 |  |  |  |  |  |  |  |
|  |  | *CcH155 | 1 |  | H149 |  |  |  |  |  |  |
|  |  | CcH166 | 28 | 2 | H152 |  |  | Cc36 |  |  |  |
|  |  | *CcH198 | 1 |  |  |  |  |  |  |  |  |
|  |  | CcH206 | 13 |  |  |  |  |  |  |  |  |
|  |  | RANH131 |  | 4 | H131, H132 |  |  |  |  |  |  |
|  |  | RANH115 |  | 5 | H115 |  |  |  |  |  |  |
| **East** | **E4** | *CcH2 | 1 |  |  |  |  |  |  |  |  |
|  |  | CcH15 | 2 |  |  |  |  |  |  |  |  |
|  |  | CcH16 | 2 |  |  |  |  |  |  |  |  |
|  |  | CcH37 | 2 |  |  |  |  |  |  |  |  |
|  |  | CcH46 | 3 |  |  |  |  |  |  |  |  |
|  |  | CcH51 | 6 |  |  |  |  |  |  |  |  |
|  |  | CcH85 | 6 |  |  |  |  |  |  |  |  |
|  |  | CcH90 | 4 |  |  |  |  |  |  |  |  |
|  |  | CcH92 | 2 | 9 | H28 |  |  |  |  |  |  |
|  |  | CcH93 | 11 |  |  |  |  |  |  |  |  |
|  |  | CcH97 | 12 |  |  |  |  |  |  |  |  |
|  |  | *CcH100 | 1 |  |  |  |  |  |  |  |  |
|  |  | CcH102 | 4 |  |  |  |  |  |  |  |  |
|  |  | *CcH107 | 1 |  |  |  |  |  |  |  |  |
|  |  | *CcH110 | 1 |  |  |  |  |  |  |  |  |
|  |  | *CcH115 | 1 |  |  |  |  |  |  |  |  |
|  |  | CcH121 | 16 | 1 | H9 |  |  | Cc33 |  |  |  |
|  |  | *CcH123 | 1 |  |  |  |  |  |  | | |
|  |  | CcH138 | 7 |  |  |  |  |  |  | |  |
|  |  | *CcH142 | 1 |  |  |  |  |  |  | |  |
|  |  | *CcH156 | 1 |  |  |  |  |  |  | |  |
|  |  | CcH162 | 3 |  |  |  |  |  |  | |  |
|  |  | CcH175 | 3 |  | H81 |  |  |  |  | |  |
|  |  | CcH182 | 41 | 7 | H30 |  |  |  |  | |  |
|  |  | CcH184 | 3 | 1 |  | L24 |  |  |  | |  |
|  |  | *CcH185 | 1 |  |  |  |  |  |  | |  |
|  |  | *CcH197 | 1 |  |  |  |  |  |  | |  |
|  |  | CcH212 | 2 |  |  |  |  |  |  | |  |
|  |  | CcH217 | 14 |  |  |  |  |  |  | |  |
|  |  | *CcH218 | 1 |  |  |  |  |  |  | |  |
| Table S1 - continued | | | | | | | | | | |  |
| **East** | **E4** | RANH119 |  | 4 | H119 |  |  |  |  | |  |
|  |  | RANH116 |  | 9 | H116 |  |  |  |  | |  |
|  |  | GENL22 |  | 2 |  | L22 |  |  |  | |  |
| **West** | **W1** | CcH50 | 4 |  |  |  |  |  |  | |  |
|  |  | CcH52 | 13 |  |  |  |  |  |  | |  |
|  |  | *CcH76 | 1 |  |  |  |  |  |  | |  |
|  |  | *CcH84 | 1 |  |  |  |  |  |  | |  |
|  |  | *CcH151 | 1 |  |  |  |  |  |  | |  |
|  |  | CcH159 | 16 |  |  |  |  |  |  | |  |
|  |  | RANH92 |  | 24 | H92 |  |  | Cc1, Cc2 |  | |  |
|  |  | RANH70 |  | 7 | H70, H77 |  |  |  |  | |  |
|  |  | RANH69 |  | 6 | H69 |  |  |  |  | |  |
| **West** | **W2** | CcH147 | 3 |  |  |  |  |  |  | |  |
|  |  | CcH203 | 2 |  |  |  |  |  |  | |  |
|  |  | *CcH221 | 1 |  |  |  |  |  |  | |  |
|  |  | RANH161 |  | 3 | H161 |  |  | Cc3 |  | |  |
|  |  | RANH160 |  | 10 | H160 |  |  | Cc6 |  | |  |
|  |  | RANH71 |  | 26 | H71 |  |  | Cc5 |  | |  |
|  |  | RANH57 |  | 2 | H57 |  |  |  |  | |  |
|  |  | RANH25 |  | 13 | H25 |  |  |  |  | |  |
|  |  | RANH6 |  | 15 | H6 |  |  | Cc4 |  | |  |
| **Siberian** | **Siberian** | CpH1 | 39 | 6 |  |  |  | Cp40 |  | |  |
|  |  | CpH2 | 2 |  |  |  |  |  |  | |  |
|  |  | *CpH3 | 1 |  |  |  |  |  |  | |  |
|  |  | *CpH4 | 1 |  |  |  |  |  |  | |  |
|  |  | *CpH5 | 1 |  |  |  |  |  |  | |  |
|  |  | CpH6 | 6 |  |  |  |  |  |  | |  |
|  |  | *CpH7 | 1 |  |  |  |  |  |  | |  |
|  |  | CpH8 | 27 |  |  |  |  |  |  | |  |
|  |  | *CpH9 | 1 |  |  |  |  |  |  | |  |
|  |  | CpH10 | 10 |  |  |  |  |  |  | |  |
|  |  | CpH11 | 2 | 2 |  |  |  | Cp41, Cp39 |  | |  |
|  |  | *CpH12 | 1 |  |  |  |  |  |  | |  |
|  |  | *CpH13 | 1 |  |  |  |  |  |  | |  |
|  |  | *CpH14 | 1 |  |  |  |  |  |  | |  |
|  |  | CpH15 | 3 |  |  |  |  |  |  | |  |
|  |  | CpH16 | 4 |  |  |  |  |  |  | |  |
|  |  | *CpH17 | 1 |  |  |  |  |  |  | |  |
|  |  | CpH18 | 4 |  |  |  |  |  |  | |  |
|  |  | CpH19 | 43 | 2 |  |  |  |  |  | |  |
|  |  | *CpH20 | 1 |  |  |  |  |  |  | |  |
|  |  | CpH21 | 1 | 1 |  |  |  | Cp38 |  | |  |
|  |  | CpH22 | 2 |  |  |  |  |  |  | |  |
|  |  | *CpH23 | 1 |  |  |  |  |  |  | |  |
|  |  | CpH24 | 2 | 1 |  |  |  | Cp43 |  | |  |
|  |  | *CpH25 | 1 |  |  |  |  |  |  | |  |
|  |  | CpH26 | 17 |  |  |  |  |  |  | |  |
|  |  | *CpH27 | 1 |  |  |  |  |  |  | |  |
|  |  | CpH28 | 82 | 1 |  |  |  |  |  | |  |
