# Supplementary material for: Pan‐European phylogeography of the European roe deer (Capreolus capreolus)
Source: Ecol Evol. 2022 May 19;12(5):e8931. doi: 10.1002/ece3.8931 (PMC9120558; doi:10.1002/ece3.8931)
Supplement: Supplementary file 2 — Table S2 [file ECE3-12-e8931-s001.docx]

Table S2. Statistics of sudden demographic expansion model fit for clades and subclades of the European lineage of roe deer (see Figure 2) described by two parameters: sum of squared deviations (SSD) and Harpending’s raggedness index (Ragg). Values of Tajima’s *D* and Fu’s *Fs* tests were marked with statistical significance levels (**p* < 0.05; ***p* < 0.01). *P*-value for test of the SSD model validity and Ragg test of goodness-of-fit are included in the brackets.

| Subclade | Tajima’s *D* | Fu’s *Fs* | Demographic expansion | |
| --- | --- | --- | --- | --- |
|  |  |  | SSD (*p*-value) | Ragg (*p*-value) |
| Central | –1.144 | –284.028** | 0.002 (0.087) | 0.014 (0.243) |
| C1 | –0.922 | –71.976** | 0.003 (0.089) | 0.020 (0.331) |
| C2 | –0.736 | –18.425** | 0.001 (0.939) | 0.011 (0.977) |
| C3 | –1.585* | –5.559** | 0.000 (0.328) | 0.582 (0.703) |
| C4 | –0.274 | –21.065** | 0.016 (0.062) | 0.320 (0.112) |
| C5 | –1.167 | 1.378 | 0.296 (0.001) | 0.519 (0.927) |
| C6 | 0.386 | –2.396* | 0.020 (0.107) | 0.081 (0.064) |
| C7 | –0.506 | –1.096 | 0.070 (0.099) | 0.237 (0.051) |
| C8 | 0.277 | –2.668* | 0.057 (0.021) | 0.139 (0.004) |
| Eastern | –1.175 | –64.797** | 0.001 (0.292) | 0.020 (0.462) |
| E1 | –1.834** | –6.776** | 0.001 (0.389) | 0.462 (0.659) |
| E2 | –0.281 | –1.793 | 0.027 (0.229) | 0.074 (0.232) |
| E3 | –0.609 | –9.773** | 0.001 (0.215) | 0.038 (0.409) |
| E4 | 1.226 | –13.784** | 0.011 (0.104) | 0.047 (0.059) |
| Western | 0.762 | –0.225 | 0.014 (0.081) | 0.039 (0.052) |
| W1 | 1.950 | 3.567 | 0.043 (0.045) | 0.065 (0.090) |
| W2 | 0.554 | 0.646 | 0.061 (0.042) | 0.123 (0.043) |
| Total | –0.927 | –34.012** | − | − |
