# Supplementary material for: Pan‐European phylogeography of the European roe deer (Capreolus capreolus)
Source: Ecol Evol. 2022 May 19;12(5):e8931. doi: 10.1002/ece3.8931 (PMC9120558; doi:10.1002/ece3.8931)
Supplement: Supplementary file 3 — Table S3 [file ECE3-12-e8931-s002.docx]

Table S3: Results of spatial and demographic expansion time calculations according to the division into clades and subclades assigned to the European lineage of roe deer (see Figure 2). Expansion time was calculated using the equation: T=τ/2 μ where μ the minimum (0.08) and maximum (0.04) mutation rate in units of substitutions per locus per generation and τ is Tau calculated with the mismatch analyses. Mean and 95% CI was calculated according to the given Tau value. The roe deer generation time was taken to be 3 years.

| Subclade  and lineage | Tau  Mean (95% CI) | Mutation rate 0.08  Mean (95% CI) | Mutation rate 0.04  Mean (95% CI) |
| --- | --- | --- | --- |
| **Spatial expansion model** | | | |
| **Central** | 5.529 (3.610-6.412) | 56 650 (37 990-65 700) | 113 300 (73 970-131 390) |
| C1 | 4.106 (2.250-5.010) | 42 070 (23 050-51 330) | 84 140 (46 110-102 660) |
| C2 | 1.866 (0.559-4.540) | 19 120 (5 730-46 520) | 38 240 (11 450-93 030) |
| C3 | 0.093 (0-1.710) | 950 (0-17 520) | 1 910 (0-35 040) |
| C4 | 4.861 (2.152-6.985) | 49 800 (22 050-71 570) | 99 610 (44 100-143 130) |
| C5 | 2.783 (0-7.047) | 28 510 (0-72 200) | 57 030 (0-144 410) |
| C6 | 3.332 (0.939-4.982) | 34 140 (9 620-51 040) | 68 280 (19 240-102 090) |
| C7 | 2.079 (0.308-4.576) | 21 300 (3 160-46 880) | 42 600 (6 310-93 770) |
| C8 | 3.098 (0.753-4.925) | 31 740 (7 720-50 460) | 63 480 (15 430-100 920) |
| **Eastern** | 3.915 (1.706-5.119) | 40 110 (17 480-52 450) | 80 230 (34 960-104 900) |
| E1 | 0.062 (0-2.096) | 635 (0-21 480) | 1 270 (0-42 950) |
| E2 | 2.027 (0.406-6.123) | 20 770 (4 160-62 740) | 41 540 (8 320-125 470) |
| E3 | 2.366 (1.481-2.748) | 24 240 (15 170-28 160) | 48 480 (30 350-56 310) |
| E4 | 3.981 (1.478-5.517) | 40 790 (15 140-56 530) | 81 580 (30 290-113 050) |
| **Western** | 6.643 (3.339-12.143) | 68 060 (34 210-124 420) | 136 130 (68 420-248 830) |
| W1 | 6.642 (3.320-11.876) | 68 050 (34 020-121 680) | 136 110 (68 030-243 360) |
| W2 | 3.529 (0.743-6.469) | 36 160 (7 610-66 280) | 72 320 (15 230-132 560) |
| **Total** | 5.416 (3.800-8.156) | 55 490 (38 930-83 570) | 110 980 (77 870-167 130) |
| **Demographic expansion model** | | | |
| **Central** | 5.395 (3.793-6.215) | 55 270 (38 860-63 680) | 110 540 (77 730-127 360) |
| C1 | 4.148 (2.588-4.969) | 42 510 (26 520-53 030) | 85 020 (50 910-101 820) |
| C2 | 3.098 (0.686-5.646) | 31 740 (7 030-57 850) | 63 480 (14 060-115 700) |
| C3 | 3.000 (0-3.500) | 30 740 (0-35 860) | 61 480 (0-71 720) |
| C4 | 5.209 (2.148-7.885) | 53 370 (22 010-80 790) | 106 740 (44 020-161 580) |
| C5 | 0 (0-0.723) | 0 (0-7 410) | 0 (0-14 820) |
| C6 | 3.531 (1.266-5.654) | 36 180 (12 970-57 930 | 72 360 (25 940-115 860) |
| C7 | 2.350 (0-5.125) | 24 070 (0-52 510) | 48 150 (0-105 020) |
| C8 | 3.143 (1.225-4.785) | 32 200 (12 551-49 027) | 64 400 (25 102-98 053) |
| **Eastern** | 3.953 (2.414-4.891) | 40 500 (24 730-50 110) | 81 010 (49 470-100 220) |
| E1 | 3.000 (0-3.500) | 30 740 (0-35 860) | 61 480 (0-71 720) |
| E2 | 3.781 (0.363-7.336) | 38 740 (3 720-75 160) | 77 480 (7 440-150 330) |
| E3 | 2.359 (1.820-2.830) | 24 170 (18 650-29 000) | 48 350 (37 300-57 990) |
| E4 | 4.213 (1.760-6.186) | 43 160 (18 030-63 380) | 86 330 (36 070-126 760) |
| **Western** | 5.945 (2.668-9.014) | 60 920 (27 340-92 360) | 121 830 (54 670-184 710) |
| W1 | 8.420 (0-97.420) | 86 270 (0-998 160) | 172 540 (0-1 996 310) |
| W2 | 4.223 (0-9.020) | 43 270 (0-92 420) | 86 530 (0-184 840) |
| **Total** | 6.236 (4.135-9.605) | 63 900 (42 370-98 410) | 127 790 (84 730-196 820) |
