# Supplementary material for: Pan‐European phylogeography of the European roe deer (Capreolus capreolus)
Source: Ecol Evol. 2022 May 19;12(5):e8931. doi: 10.1002/ece3.8931 (PMC9120558; doi:10.1002/ece3.8931)
Supplement: Supplementary file 4 — Figures S1‐S9 [file ECE3-12-e8931-s003.docx]

## Figure S1


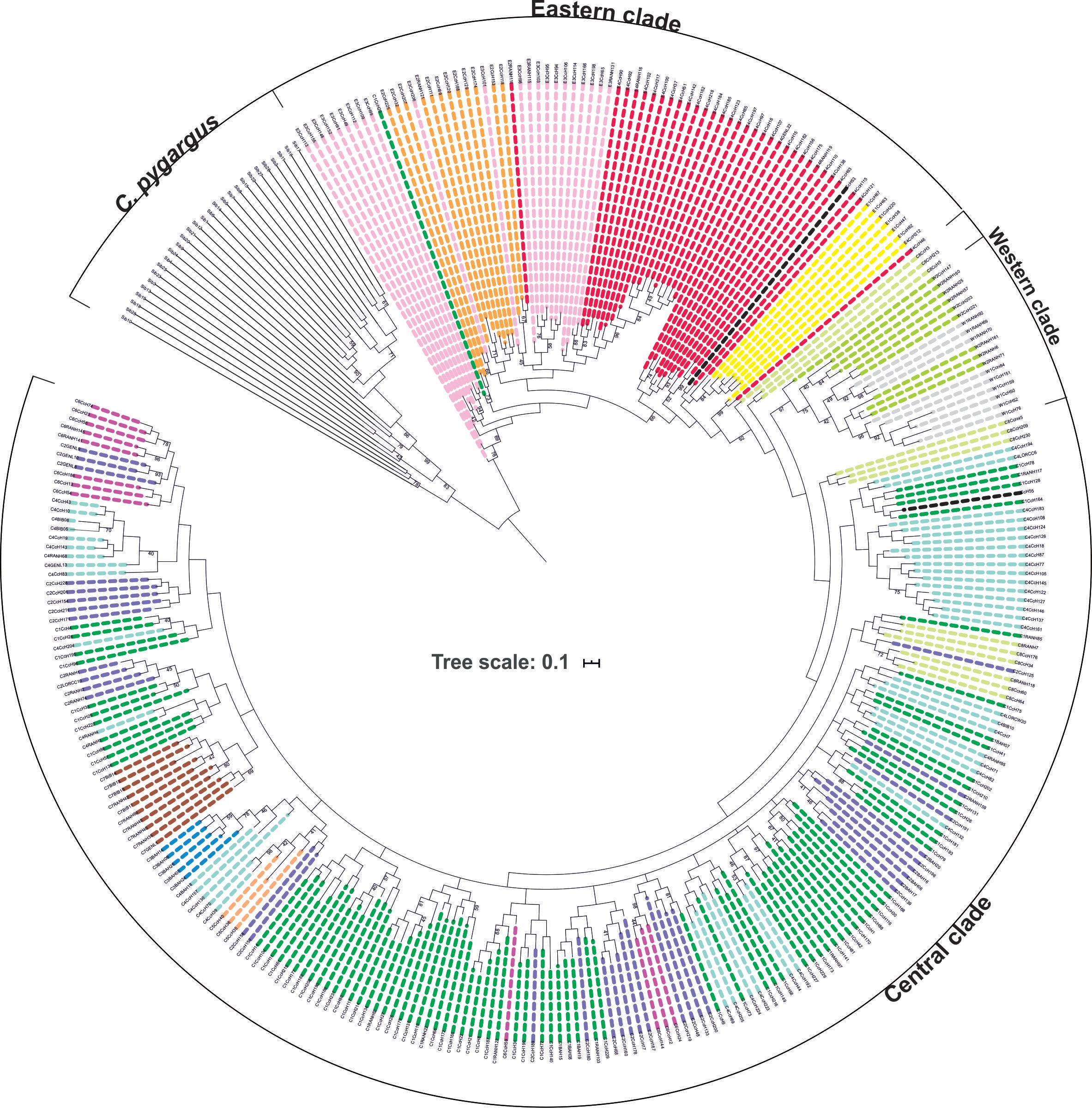


FIGURE S1: Phylogenetic Bayesian tree showing the genetic relationships among mtDNA haplotypes of the European roe deer (*Capreolus capreolus*). Division into clades follows classification proposed by Randi et al. (2004). Tree constructed in programme MrBayes3 under Hasegawa, Kishino and Yano (HKY) model. Details on haplotypes in Table S1. Colours refer to haplogroups shown in Figure 2.

**Figure S2**


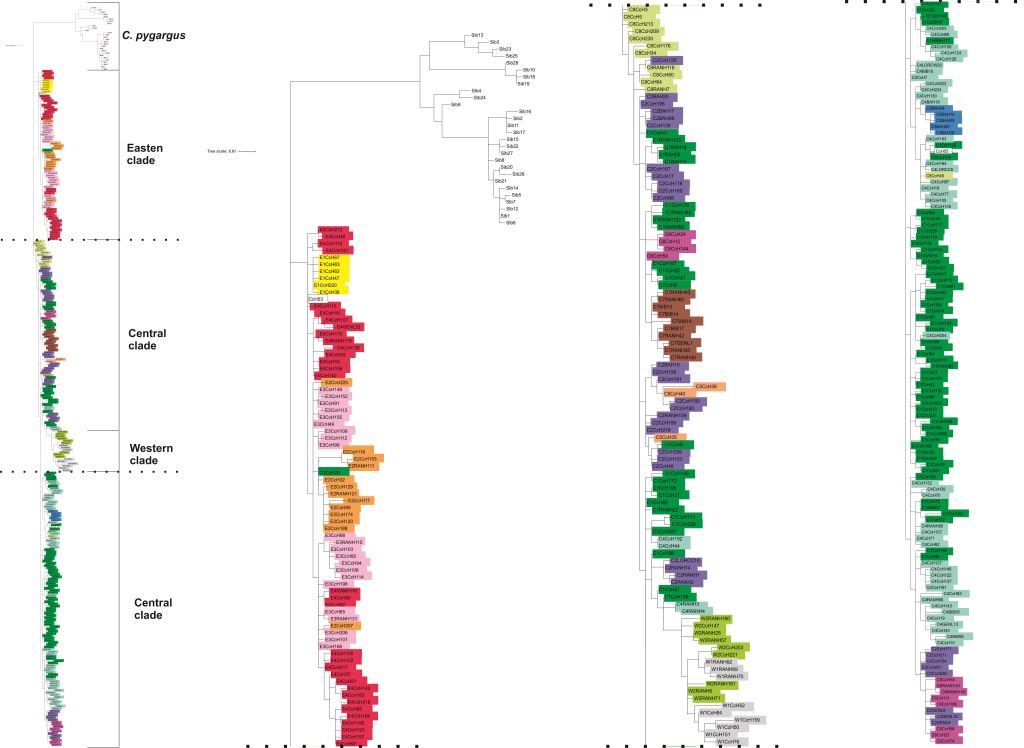


FIGURE S2: Phylogenetic tree representing the genetic relationships among indicated mtDNA haplotypes of the European roe deer (*Capreolus capreolus*). Division into clades follows classification proposed by Randi et al. (2004). Tree constructed in programme Mega 7.0. under Hasegawa, Kishino and Yano (HKY) model. First column contains the overall tree, whereas the following ones are closer looks at single portions – division marked with the dashed lines in the first column. Details on haplotypes in Table S1. Colours refer to haplogroups shown in Figure 2.

**Figure S3**


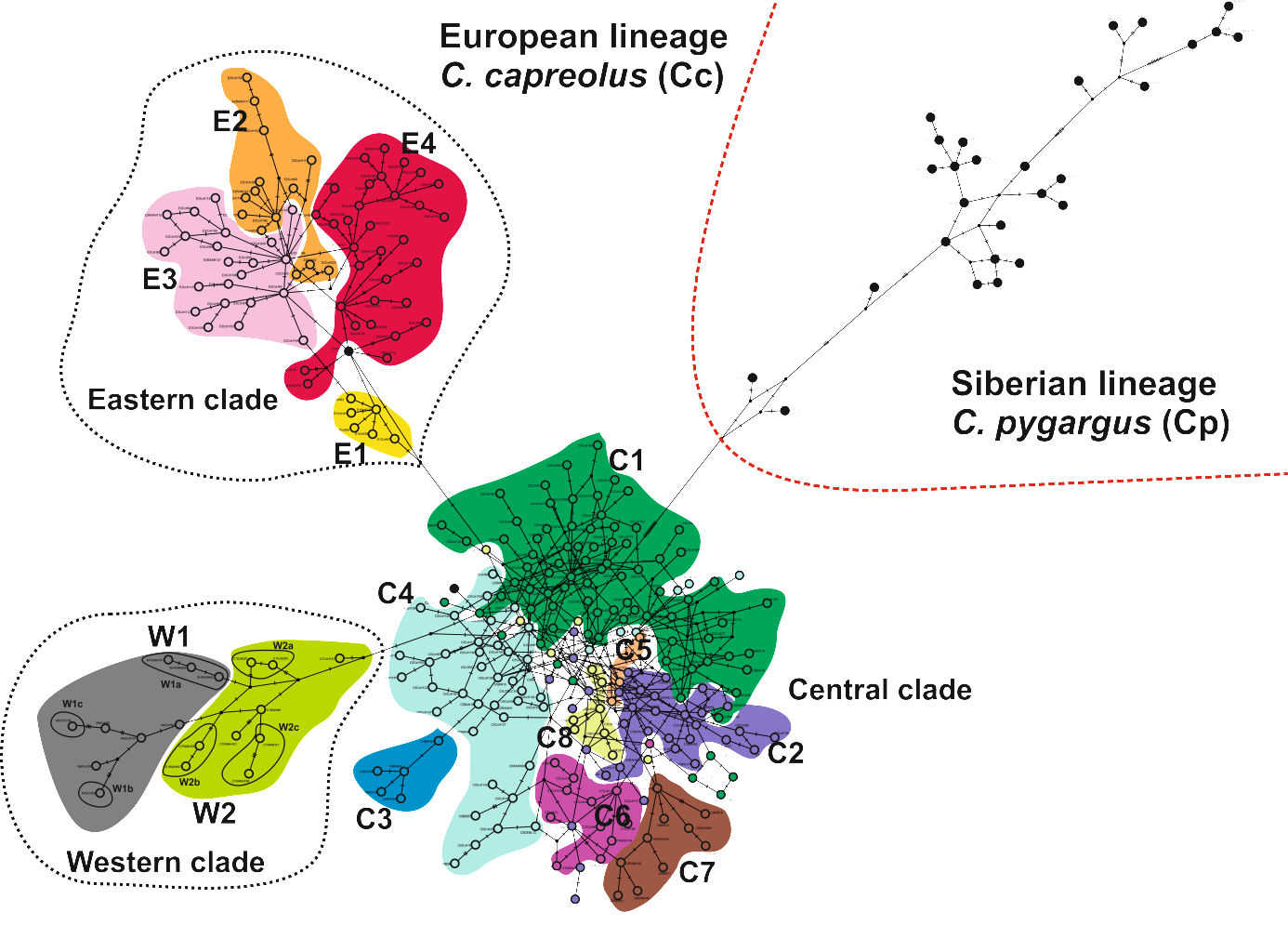


Figure S3: Phylogenetic median-joining network presenting the genetic relationship among mtDNA haplotypes of the European roe deer (*C. capreolus*). Open circles represent unique haplotypes. Small dots show missing haplotypes. Individual colours denote different groups indicated by HapView software (see Fig. 2). Division into clades follows the names previously defined by Randi et al. (2004)..

**Figure S4**

**
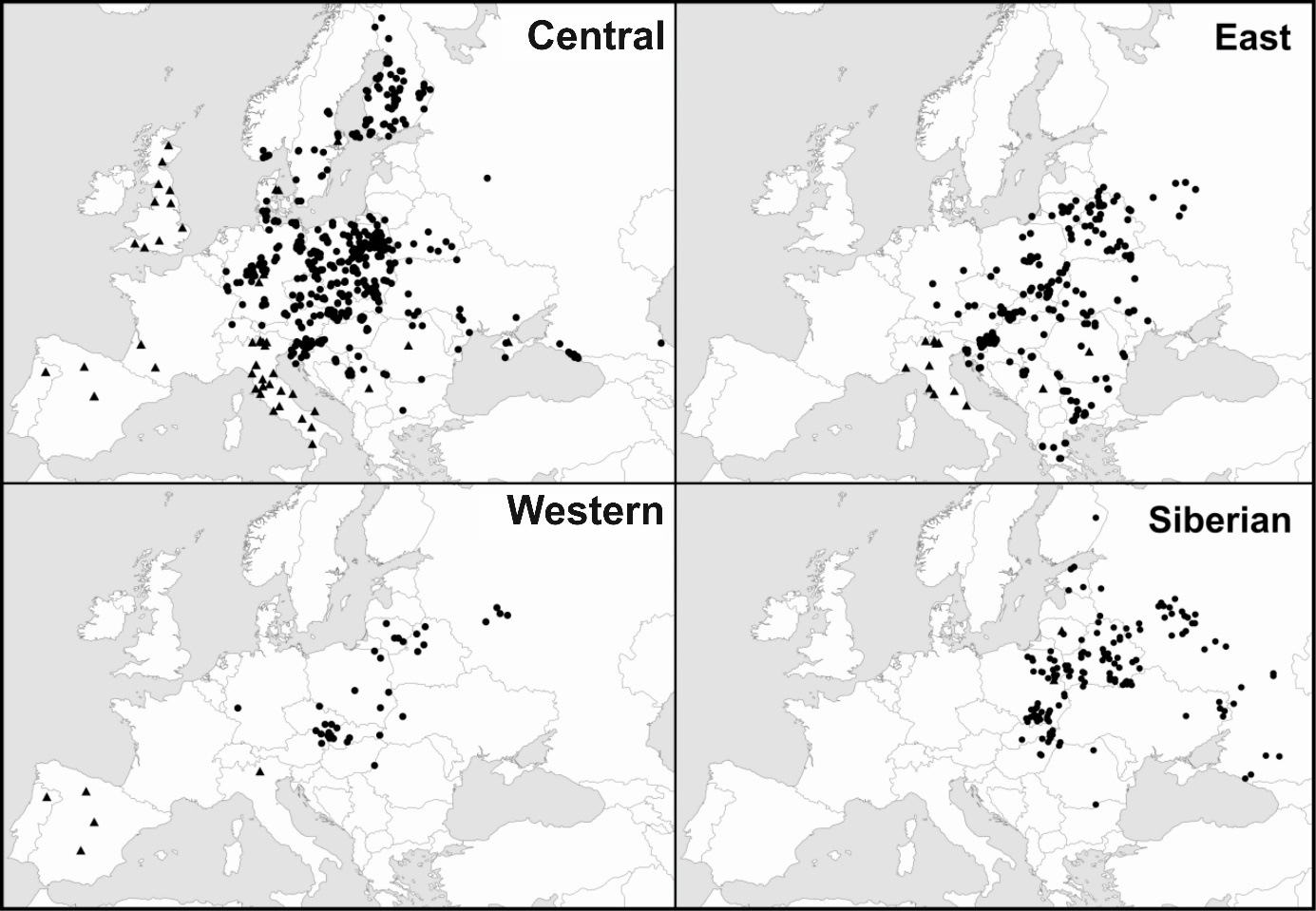
**

Figure S4: Distribution of the mtDNA Siberian lineage and three clades of the European lineage (Central, Eastern, and Western) of roe deer in Europe. Dots indicate original samples collected for this study, triangles represent literature data.

**Figure S5**

**
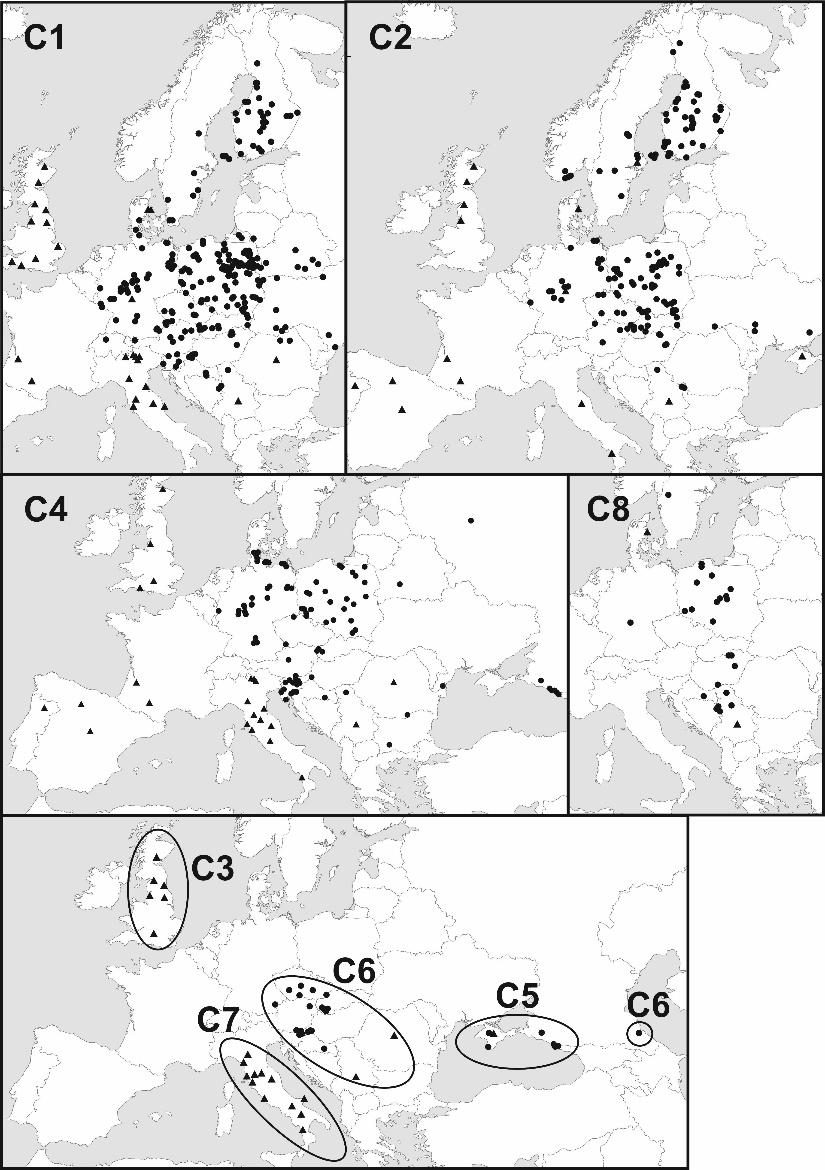
**

Figure S5: Distribution of mtDNA haplogroups C1-C8 belonging to the Central clade of the European roe deer. Division into haplogroups was based on haplotype genealogy constructed in HapView (see Fig. 2). Dots indicate original samples collected for this study, triangles represent literature data.

**Figure S6**

**
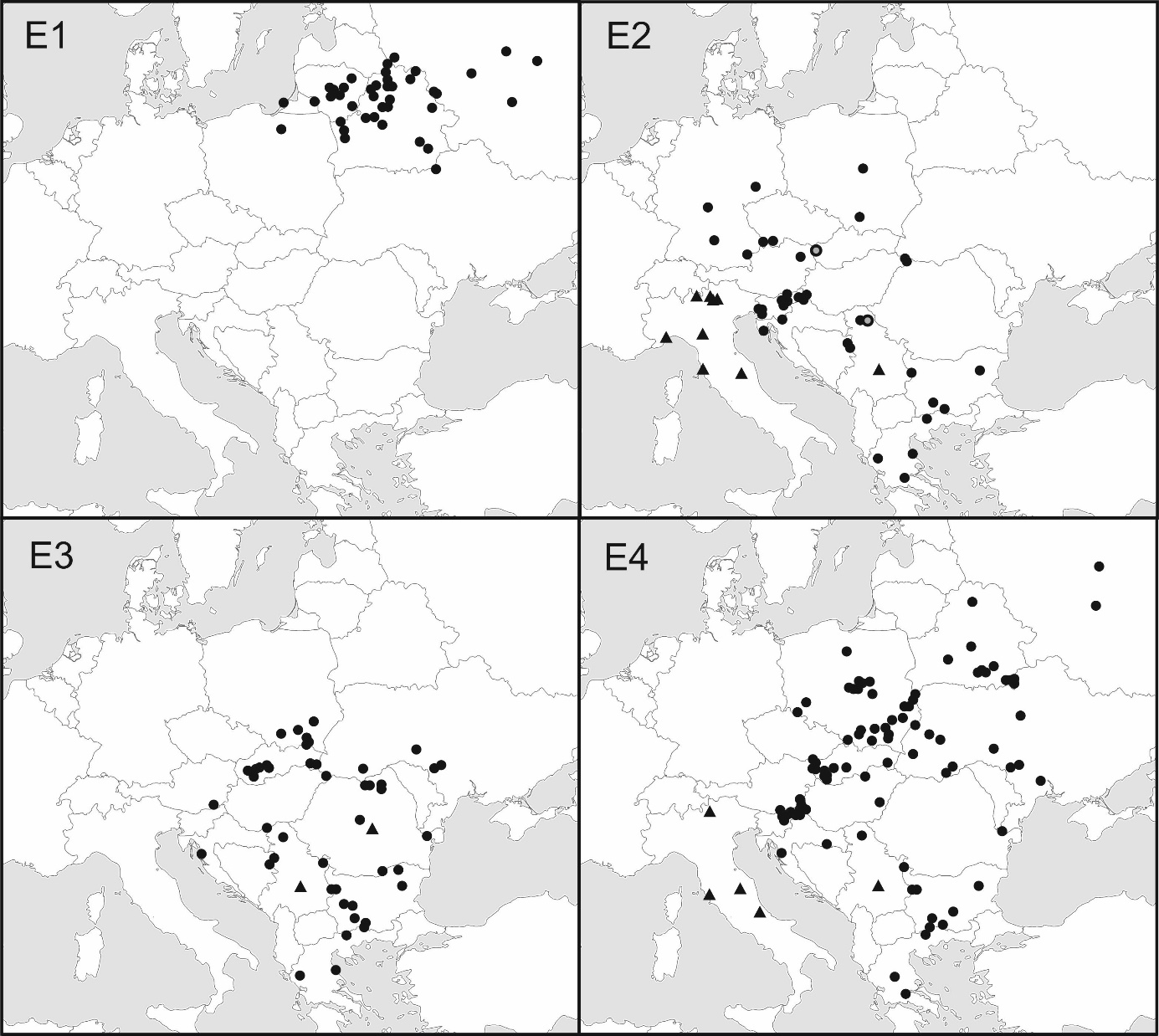
**

Figure S6: Distribution of mtDNA haplogroups E1-E4 belonging to the Eastern clade of the European roe deer. Grey points (in E2 panel) correspond to an ungrouped haplotype CcH53. Other explanations as in Fig. S5.

**Figure S7**

**
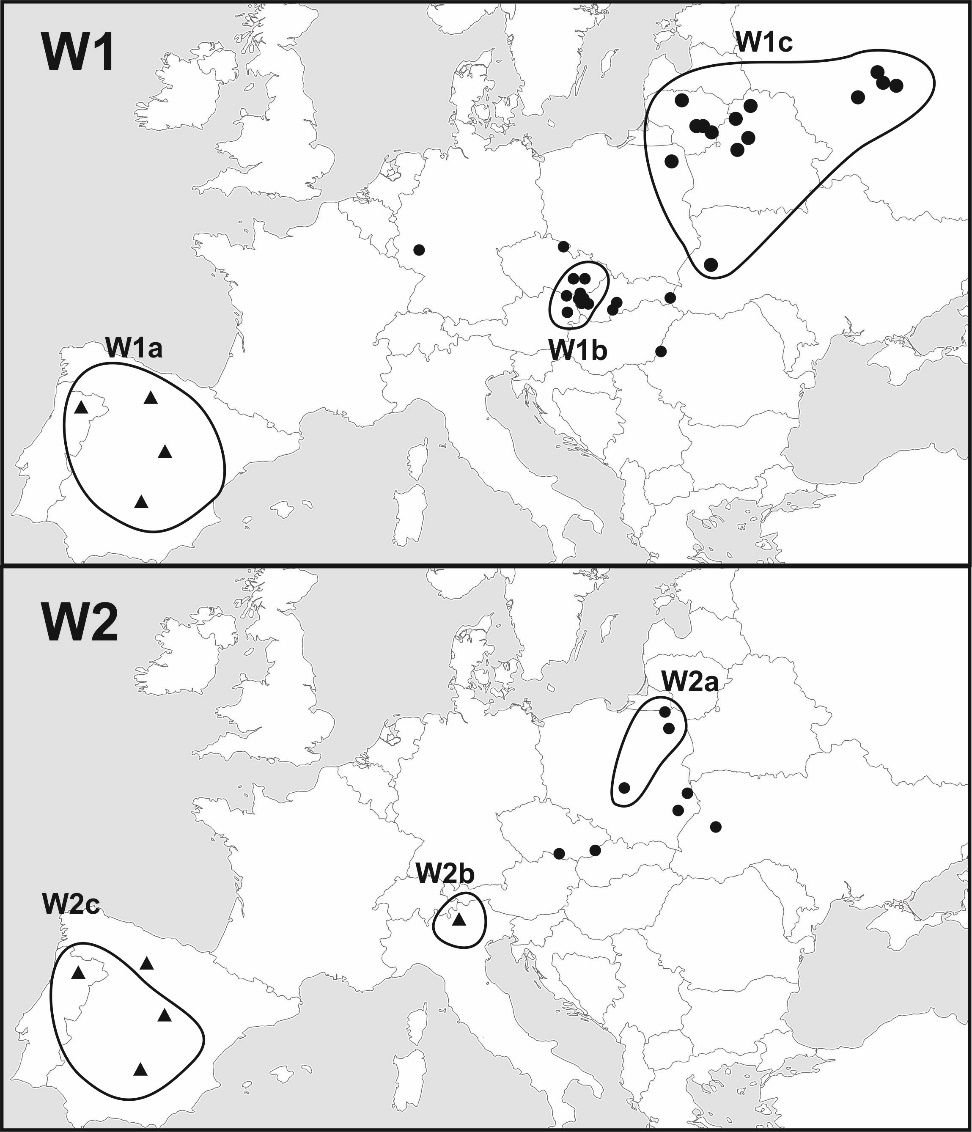
**

Figure S7: Distribution of mtDNA haplogroups W1 and W2 belonging to the Western clade of the European roe deer. Black lines indicate ranges of haplotype subgroups defined within W1 and W2 (marked a, b, c in Fig. 2). Other explanations as in Fig. S5.

**Figure S8**

**
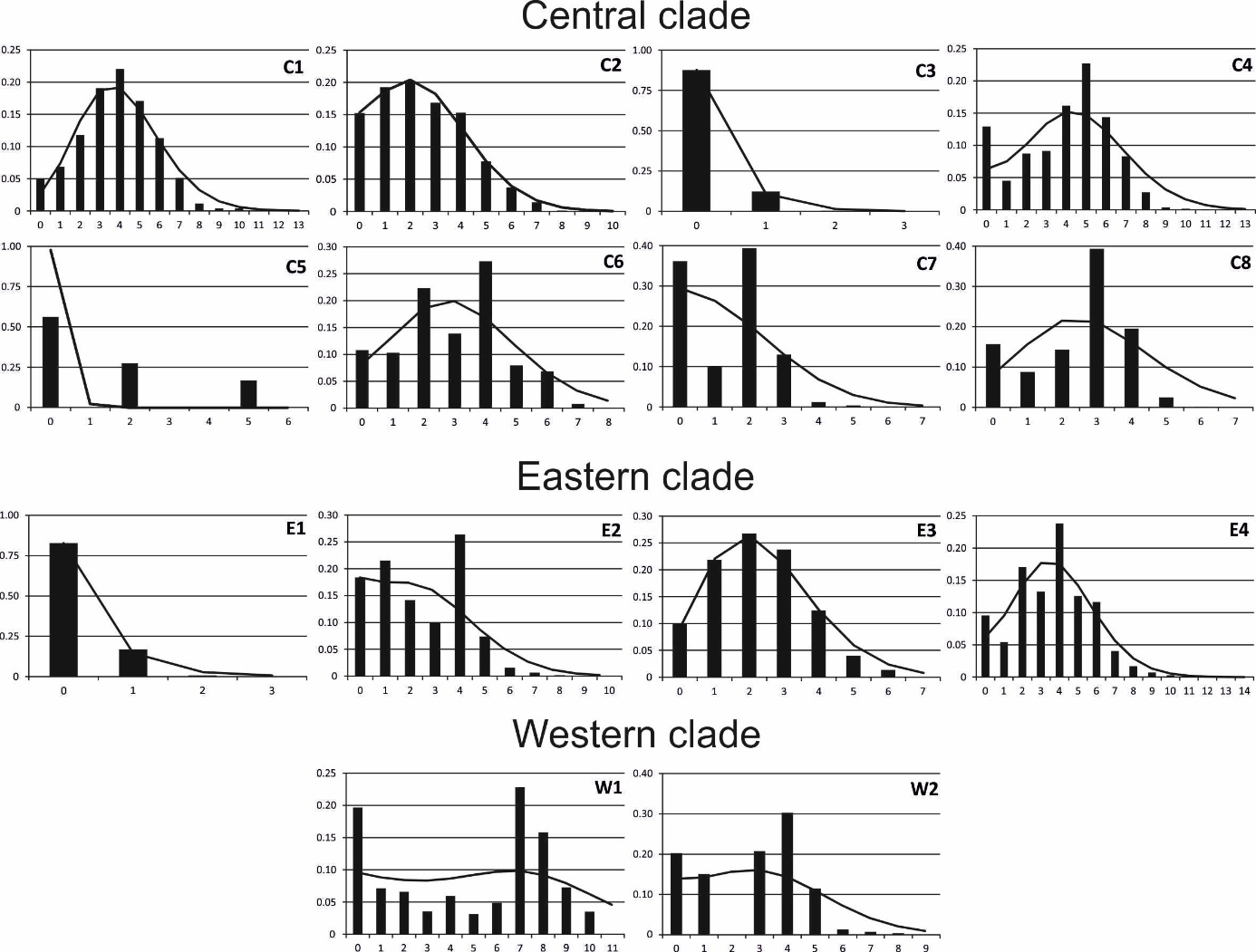
**

FIGURE S8: Mismatch distribution. Graphs represent the mismatch distribution for the all haplogroups among three main European roe deer clades (Central, Eastern and Western) found in the investigated population. Bars represents observed frequency of pairwise distributions, while dashed lines correspond to the frequency expected based on the sudden expansion model.

**Figure S9**


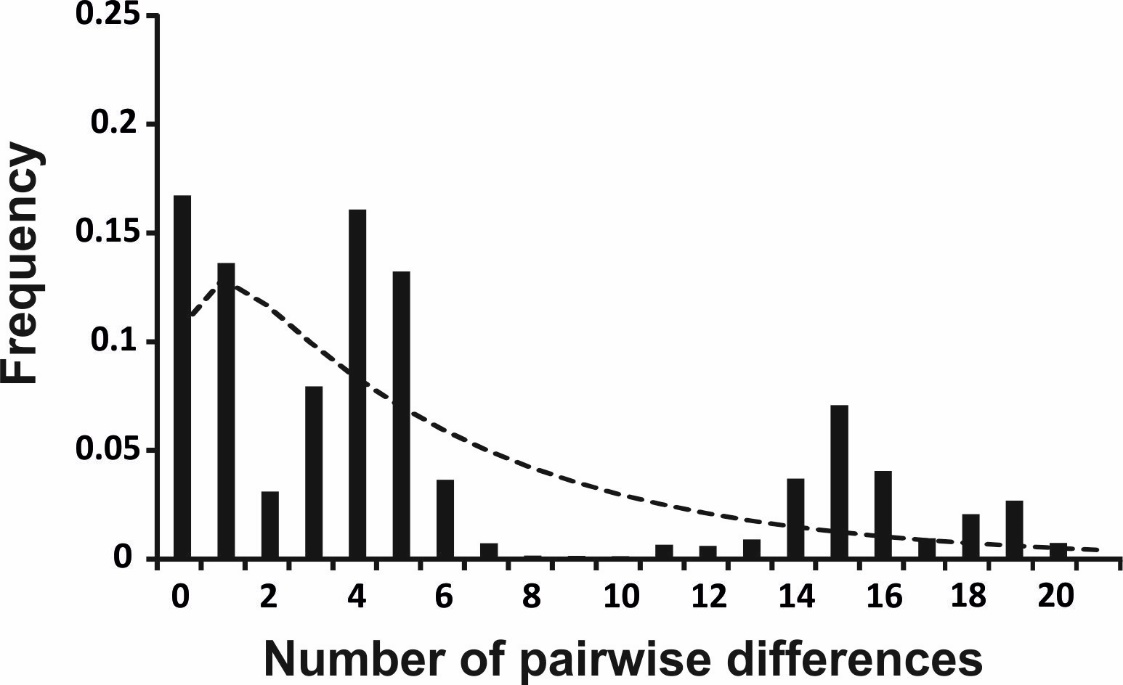


FIGURE S9: Mismatch distribution. Graph represents the mismatch distribution for the Siberian roe deer lineage found in the investigated population. Bars represents observed frequency of pairwise distributions, while dashed lines correspond to the frequency expected based on the sudden expansion model.
